# A Green Approach for Allylations of Aldehydes and Ketones: Combining Allylborate, Mechanochemistry and Lanthanide Catalyst

**DOI:** 10.3390/molecules21111539

**Published:** 2016-11-16

**Authors:** Viviane P. de Souza, Cristiane K. Oliveira, Thiago M. de Souza, Paulo H. Menezes, Severino Alves, Ricardo L. Longo, Ivani Malvestiti

**Affiliations:** Departamento de Química Fundamental—Universidade Federal de Pernambuco, Av. Jornalista Aníbal Fernandes, s/n—Cidade Universitária, Recife—PE 50740-560, Brazil; psouza.viviane@gmail.com (V.P.d.S.); ckoliveira@gmail.com (C.K.O.); tmsmuniz@gmail.com (T.M.d.S.); pmenezes@ufpe.br (P.H.M.); salvesjr@ufpe.br (S.A.J.); longo@ufpe.br (R.L.L.)

**Keywords:** potassium allyltrifluoroborate, mechanochemistry, selectivity, alcohols, catalysis, lanthanide

## Abstract

Secondary and tertiary alcohols synthesized via allylation of aldehydes and ketones are important compounds in bioactive natural products and industry, including pharmaceuticals. Development of a mechanochemical method using potassium allyltrifluoroborate salt and water, to successfully perform the allylation of aromatic and aliphatic carbonyl compounds is reported for the first time. By controlling the grinding parameters, the methodology can be selective, namely, very efficient for aldehydes and ineffective for ketones, but by employing lanthanide catalysts, the reactions with ketones can become practically quantitative. The catalyzed reactions can also be performed under mild aqueous stirring conditions. Considering the allylation agent and its by-products, aqueous media, energy efficiency and use of catalyst, the methodology meets most of the green chemistry principles.

## 1. Introduction

Allylations of carbonyl compounds are important synthetic tools because they form homoallylic alcohols that are fundamental building blocks for many natural products, including bioactive molecules as well as pharmaceuticals [1,2,3]. Therefore, many methods using different allylation agents, reaction media and activation process have been and are still being developed or improved for these transformations [1,2,3,4,5,6,7,8,9]. For instance, allyl-stannanes, -silanes, and -boranes are some of the allylation agents being explored [1,2,3,4,5,6,7,10,11,12,13] as well as Barbier-type allylation using different metals, e.g., Zn, In, Sn, Mn, Sb, Mg, Bi, and Al [9]. In addition, distinct activation or mediated processes have been employed, such as ultrasound [14,15], microwave [16,17,18,19], mechanochemistry [20,21], heterogeneous catalysts [22,23,24,25,26], and phase transfer [27,28], as well as green media (water) and even solvent-free processes [29,30,31]. Most of the recent developments attempt to target less reactive carbonyl compounds such as ketones, and are steered toward greener processes, especially by reducing or eliminating hazardous by-products, (halide) solvents, expensive and demanding separation processes, and by employing greener and cheaper catalysts [4,5,6]. Mechanochemical methods are solid state processes that employ mechanical energy to induce chemical reactions and transition phase changes [32,33,34]. Thus, they have great potential to provide greener processes [35] and have already been employed in the allylations of aldehydes by allyltributylstannane catalyzed by phosphotungstic acid under solvent-free conditions upon grinding with a pestle in a mortar [20] and in the Barbier-type allylations of aromatic carbonyl compounds by allyl halide mediated by bismuth under solvent-free conditions upon milling with stainless steel balls in a stainless steel vial [20]. We report now the mechanochemical allylation of aromatic and aliphatic carbonyl compounds by potassium allyltrifluoroborate, which is a greener agent because it generates inert, nontoxic, water soluble salts that are easily removed from the reaction medium. It is noteworthy that this method employs water as liquid assisted grinding (LAG) or as solvent and its selectivity for aldehydes in the presence of ketones can be controlled by the grinding parameters. This methodology can be improved by using lanthanide catalysts that is able to perform allylation of ketones almost quantitatively. This makes this methodology quite efficient, versatile, selective and green.

## 2. Results

The allylation of *p*-nitrobenzaldehyde by potassium allyltrifluoroborate using mechanochemistry was initially explored to provide insights into the effects of the milling parameters, especially, milling equipment, LAG, and solvent. The scope of the mechanochemical synthesis was explored by using several substituted benzaldehydes with reaction yields ranging from good to excellent depending upon the physical state of the substrate and the nature of the substituents. Controlling of the milling parameters allows the mechanochemical method to be specific for aldehydes and it was ineffective for allylation of ketones. On the other hand, increasing the kinetic energy of the grinding process and the reaction times led to a successful allylation of acetophenone. This reaction was made much more efficient by using lanthanide catalysts with excellent to quantitative yields obtained via mechanochemistry, which also worked fairly well via aqueous solution stirring.

### 2.1. Mechanochemical Allylation of p-Nitrobenzaldehyde: Solvent Effects

**Scheme 1:**
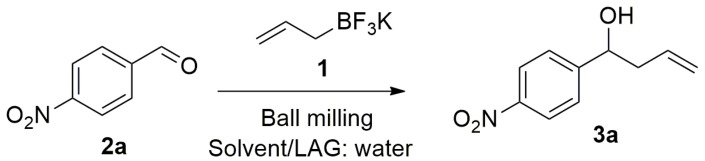
Mechanochemical allylation of *p*-nitrobenzaldehyde (**2a**) by potassium allyltrifluoroborate (**1**).

Because this is a pioneer study of aldehyde allylation by allyltrifluoroborate via mechanochemistry, several experiments were needed to set the parameters and variables of the procedure, especially the solvent. The *p*-nitrobenzaldehyde substrate was chosen because it is a suitable model for allylations and it has been previously employed in several different procedures. After some preliminary tests with a simple milling equipment designed for mechanical cell or tissue disruption, the reaction was practically quantitative after 20 min milling at room temperature. Table 1 (and Scheme 1) presents the main results and shows the effects of the solvent, especially, of water.

The results in Table 1 clearly shows the need of an aqueous medium probably to activate the allylation agent. These observations prompted a new procedure in two steps and one pot (experiment 7 in Table 1), where potassium allyltrifluoroborate is milled with small amounts of water for 10 min, followed by the addition of the aldehyde and 10 min milling.

### 2.2. Mechanochemical Allylation of Benzaldehydes: Substituent Effects

**Scheme 2:**
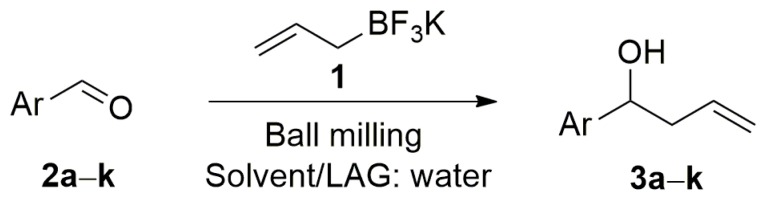
Mechanochemical allylation of arylaldehydes (**2a**–**k**) by potassium allyltrifluoroborate (**1**) yielding homoallylic alcohols (**3a**–**k**). Ar = 4-NO_2_-C_6_H_4_, 3-NO_2_-C_6_H_4_, 2-NO_2_-C_6_H_4_, 4-Cl-C_6_H_4_, 4-Br-C_6_H_4_, 2-OMe-C_6_H_4_, 3-OMe-C_6_H_4_, 4-OMe-C_6_H_4_, 2-Me-C_6_H_4_, 1-naftyl, and 2-naftyl.

To explore the scope of this process (two steps-one pot) as well as the electronic effects of the substituents on the reaction yields, Table 2 (and Scheme 2) presents the allylation of arylaldehydes, including benzaldehydes substituted in different positions by electron withdrawing and donating groups.

The results in Table 2 seem to suggest that electron-donating substituents decrease the reactivity of the substrate. However, in mechanochemical processes, the physical state of the reagents is also relevant and it can be observed that the lower yields were obtained for liquid substrates.

### 2.3. Mechanochemical Allylation of Benzaldehydes: Effects of the Grinding Parameters

**Scheme 3:**
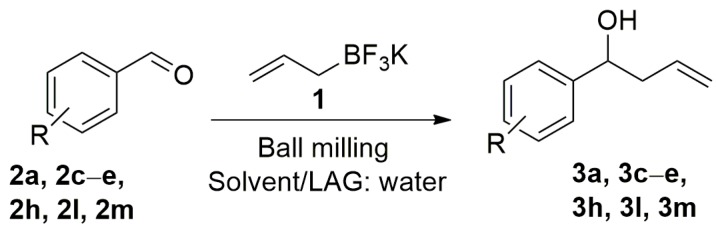
Mechanochemical allylation of substituted benzaldehydes by potassium allyltrifluoroborate (**1**). R = 4-NO_2_ (**2a**), 3-NO_2_ (**2c**), 4-Cl (**2d**), 4-Br (**2e**), 4-MeO (**2h**), 3-OH (**2l**), and 4-OH (**2m**).

These results show that even with a simple grinding equipment (BioSpec bead impact-shaken vessel Mini Bead Beater-1 model), usually employed in mechanical cell disruption and tissue homogenization, the allylation of nitrobenzaldehyde can be quantitative. However, in order to scale up the process, as well as to test different grinding parameters (e.g., frequency, grinding medium—ball bearings and jars), this available equipment is not adequate and a vibratory ball mill equipment was employed. Table 3 (and Scheme 3) presents the results of allylations of substituted benzaldehydes using a one-step procedure with a Retsch MM200 model mill equipped with a stainless steel 5 mL vial and two 10 mm stainless steel balls operating at 25 Hz.

The results obtained in Table 2 using the BioSpec equipment (two-steps one pot procedure) are fairly well reproduced by the Retsch MM200 mill (one-step procedure) in Table 3, where it is also observed that liquid substrates lead to smaller reaction yields. Noticed that a significant decrease of the milling frequency (from 70 Hz to 25 Hz) has not affect the reaction yields, suggesting that the grinding medium is more relevant. As a result, a milling auxiliary, SiO_2_(s) or NaCl(s), was employed for the liquid substrates in an attempt to improve the reaction yields, using the *p*-nitrobenzaldehyde as control. It is observed (entries 8–11 in Table 3) that the reaction yields decreased considerably in the presence of the grinding auxiliary.

### 2.4. Mechanochemical Allylation of Acetophenone: Effects of the Grinding Parameters, Solvent, and Catalyst

Aiming at expanding the scope of the one-step mechanochemical method to other carbonyl substrates the allylation of acetophenone was performed under different grinding conditions and in the presence of the europium catalyst MandEu.

**Scheme 4:**
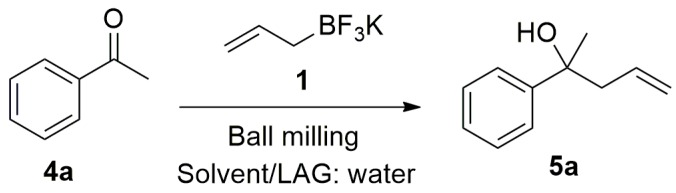
Mechanochemical allylation of acetophenone (**4a**) by potassium allyltrifluoroborate (**1**).

Table 4 (and Scheme 4) presents the reaction yields using the Retsch MM200 mill (one-step procedure) with an Eppendorf jar and glass beads.

Table 5 presents the reaction yields using the Retsch MM200 mill (one-step procedure) with a stainless steel jar and stainless steel balls.

The results in Table 4 indicate that under lower mechanical energy conditions (polymer jar and glass beads), the allylation is selective for aldehydes (Table 2) and ineffective for acetophenone on a time scale of 20 min. The reaction yields for the tertiary alcohol increase to ca. 70% in the presence of the lanthanide catalyst (MandEu) and water. Increasing the mechanical energy (stainless steel jar and stainless steel balls) favors the allylation of acetophenone even in the absence of catalyst; however, it degrades the allyltrifluoroborate or its activated derivatives. Thus, adding the same amount of the allylation agent separated into three portions in 30 min intervals leads to a significant increase of the reaction yield and becomes quantitative under catalytic conditions (Table 5).

### 2.5. Allylation of Ketones in Solution: Control Experiments and Catalysis

In an attempt to separate the effects of the mechanochemistry from that of the catalyst, the allylation of acetophenone was performed in solution under constant stirring. Table 6 presents the results for both europium catalysts (EuFum and MandEu). The results in Table 6 show that the MandEu catalyst is quite efficient for promoting the allylation of acetophenone by potassium allyltrifluoroborate. Notice, however, the need of (halide) organic solvent because of solubility problems of the reaction components. The EuFum catalyst is an extended structure with chemical formula [Eu_2_(Fum)_3_(H_2_O)_4_·3H_2_O]_n_, Fum = fumarate (*trans*-^−^OOC–CH=CH–COO^−^), whereas MandEu is most likely a monomeric structure with chemical formula [Eu(Mand)_3_(H_2_O)_2_], Mand = mandelate (C_6_H_5_–CH(OH)–COO^−^).

### 2.6. Mechanochemical Allylation of Ketones: Scope and Reactivity

**Scheme 5:**
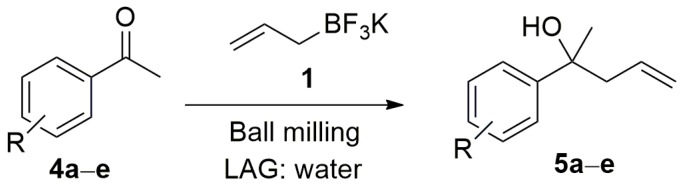
Mechanochemical allylation of substituted acetophenones (**4a**–**e**) by potassium allyltrifluoroborate (**1**). R = H, 4-NO_2_, 4-OH, 2-OMe, 4-OMe.

The scope of the methodology developed for the allylation of aromatic ketones under mechanochemical and catalytic conditions was explored for substituted acetophenones (Scheme 5 and Table 7).

The results in Table 7 indicate a significant effect of the substituents on the reaction yields probably due to their physical state and side reactions.

### 2.7. Mechanochemical Allylation of Aldehydes and Ketones: Widening the Scope and Probing the Reactivity

The results are very encouraging and prompted us to broaden the scope of the methodology to aliphatic aldehydes and ketones, which are presented in Table 8 and compared to benzaldehyde and 4-CF_3_-benzaldehyde.

It is noteworthy that the same methodology provided nearly quantitative yields for aliphatic aldehydes (heptanal and cyclohexanecarbaldehyde) and in the presence of catalyst and longer milling times the allylation of cyclohexanone was obtained in excellent yield. An acyclic aliphatic ketone (butanone) was also successfully alkylated in good conversion yield. In order to quantify the reactivity of the aromatic substrates, competitive experiments between excesses of substituted and unsubstituted aromatic carbonyl compounds were performed and the ratios between the products are presented in Table 9.

From Table 9 it can be observed that substituted and unsubstituted aromatic carbonyl compounds have similar reactivity. Competitive mechanochemical allylation of acetophenone **4a** and butanone **4g** was performed and provide 1.2 for the ratio between the areas in the GC of the products **5a** and **5g**. Similarly, two competitive experiments were performed between benzaldehyde **2n** and heptanal **2p** yielding ratios of 1.0 and 1.2 between the areas in the GC of the products **3n** and **3p**.

In addition, competing experiments between sub-stoichiometric quantities of aromatic aldehydes and aromatic ketones with potassium allyltrifluoroborate were performed under the usual milling conditions in the absence and in the presence of catalyst. For example, when a mixture of benzaldehyde (0.1 mmol), acetophenone (0.1 mmol) and potassium allyltrifluoroborate (0.11 mmol) was milled for 30 min, only the product from the allylation of the aldehyde was observed in nearly quantitative yield. The same result was obtained when excess of acetophenone (0.2 mmol) was employed or when 10 mol % MandEu was added to the mixture. Only after milling for 3 h in the presence of 10 mol % MandEu catalyst, the product from the allylation of the ketone was observed in yields smaller than those reported for the individual experiments.

## 3. Discussion

Allyltrifluoroborate salts are interesting allylation agents because they are readily available and stable under normal conditions; however, they are inert to carbonyl compounds and thus require activation, for instance, by an external Lewis acid [7]. Other boron-based allylation agents such as allylboranes, allylboronates, allylboronic esters, allylpinacolborate, and allylboronic acids are more reactive and may be used without activation. However, they are usually unstable to air and moisture and thus cannot be easily handled and purified [7,13,36,37]. In fact, allylboronic acids are so unstable that they could not be isolated and studied in a pure form until recently [37] and then were explored as allylation agents of ketones [37]. Alternatively, allyltrifluoroborates could be activated in situ by, for instance, (partial) hydrolysis leading to more reactive intermediates such as allyl(fluoro)boronic acids. Indeed, the hydrolysis of potassium organotrifluoroborates to the corresponding boronic acids is known to occur in mild aqueous silica-gel media [24,38,39]. Given the importance of water in the mechanochemical allylation of benzaldehydes presented in Table 1 and Table 2, including the success of the two-step procedure, milling of potassium allyltrifluoroborate and water followed by milling with the aldehyde, we propose that the (partial) hydrolysis of the allyltrifluoroborate, induced by the mechanical energy, to the corresponding reactive allyl(fluoro)boronic acids is a viable mechanism to describe this allylation method. A detailed analysis and exploration of this mechanism is beyond the scope of this contribution because the kinetics of this activation step via mechanochemistry is not known and would probably require in situ measurements.

Compared to the two other mechanochemical methods available for the allylation of aromatic carbonyl compounds [20,21], the present approach may be considered greener because it does not employ or generate any (organo)metallic species. The allylation agent and its by-products are stable, nontoxic and water solution salts that may require minimum or none treatment before being discarded. In addition, the present mechanochemical method can be efficiently performed in a two steps procedure, which lends flexibility to the methodology because in the second step another component(s), such as additive, catalyst, grinding auxiliary, etc., can be added to improve reaction yields and selectivity. In fact, we are currently exploring the use of solid grinding auxiliaries in the second step of this two-step procedure to improve the reaction yields of liquid substrates, because the use of NaCl or SiO_2_ auxiliary grinding in the single-step procedure decreased significantly the reaction yields (Table 3), probably because of dilution effects.

The present mechanochemical method is quite versatile and robust because by changing some of the milling parameters, specifically the frequency and grinding medium (jar and ball), the mechanical energy can be controlled and the method can be made specific for the allylation of aldehydes (small mechanical energy) in a 20 min time scale, whereas by increasing the mechanical energy the method becomes successful in the allylation of ketones in a 3 h time scale. Indeed, the methodology developed for the BioSpec mill (at 70 Hz), which was shown to be equivalent to the MM20 mill (at 25 Hz), is quite interesting because this equipment is robust, simple and quite affordable, especially with the Eppendorf jars and glass balls. However, this equipment has not been explored in mechanochemical organic reactions. In addition, this is the first example of a metal-free mechanochemical allylation of ketones. The reaction yields of the tertiary alcohol products are good and can be made quantitative by using the lanthanide-based MandEu catalyst. Because there are lesser methods for allylation of ketones, the mechanochemical method with the MandEu catalyst was further explored and shown to be very effective for several substituted acetophenones. The results showed that electron-donating substituents decrease the reaction yields. Comparisons the different results under different condition and parameters suggest that the mechanical energy is probably responsible for activating the allyltrifluoroborate reagent and the MandEu catalyst is more likely relevant for activating the carbonyl compound [25]. However, a detailed investigation of these mechanisms is beyond the scope of the present contribution, but the results are very encouraging to pursue such studies.

It is noteworthy that the lanthanide-based catalysts (EuFum and MandEu) are also effective in the allylation of ketones under mild aqueous solution stirring, without mechanochemical assistance. Under these conditions, the catalyst may have a dual role, namely, catalyzing the (partial) hydrolysis of the allyltrifluoroborate into more reactive species and to activate the carbonyl compound. Notice that these catalysts have distinct activities (Table 6), with MandEu being much more efficient. This may probably be due to their different structures, where EuFum has a metal-organic framework (MOF) structure [25,40,41,42], whereas MandEu is most likely a molecular octacoordinated complex [43,44,45]. The latter is dispersed more efficiently in solution and provide more active sites.

The methodologies developed for aromatic carbonyl compounds also work very well for the allylation aliphatic aldehydes and ketones, providing nearly quantitative conversions. The selectivity allylation of aldehydes in the presence of ketones can be achieved by controlling the milling time or the presence/absence of catalyst.

The greenness of the process is also an important issue because the methodology does not require organic solvent during the milling process and only water was used as LAG. The materials, however, became adhered to the walls of the jar and their mechanical removal was difficult and, at the scale employed, losses were unavoidable. Thus, upon completion, silica gel was added and milled for an additional 2 min, which removed the materials from walls and impregnated the silica gel. The resulting material was added directly onto a chromatography column or a minimum amount of organic solvent was used to extract the product. The water soluble salts can also be separated into a nontoxic aqueous solution.

In addition, competitive experiments under mechanochemical conditions were performed for the first time to assess the reactivity aromatic carbonyl compounds. It can be shown that performing competitive experiment with large excesses of substituted and unsubstituted substrates with respect to the allylating agent (e.g., 5:5:1), the ratio between the rate constants $k_{\mathrm{unsub}}/k_{\mathrm{sub}}$ can be approximately given by the ratio between the areas, $A_{\mathrm{unsub}}/A_{\mathrm{sub}}$, under the curves of the corresponding products in the gas-chromatogram [46]. This analysis assumes that the determining step is the actual reaction between the allylating species and the carbonyl substrate. However, under mechanochemical conditions care must be exercised because the access to the allylating species may the determining step in the reaction mechanism. The conditions employed in the competitive experiments were 0.5:0.5:0.1:0.4 mmol for the molar ratios between the competing carbonyl substrates, potassium allyltrifluoroborate, and water. It is relevant to realize that some amount of the water is involved in hydrolysis of the allyltrifluoroborate to generate the active allylating agent, possibly allyl(fluoro)boronic acid derivatives. As a result, there should be some competition between the carbonyl substrates for the allylating agent, which could become the determining step. Given the sub-stoichiometric relationship between the substrates and water, the differences in their bulk solubility in water may not be a suitable parameter to rationalize the trends in these competitive experiments. Another relevant factor is the physical states of the substrates. From Table 9 it is clear that performing competitive experiment with a solid substrate such as 4-nitrobenzaldehyde or 4-nitroacetophenone (σ_p_ = 0.78) [47] and a liquid one (benzaldehyde or acetophenone) provides unreliable results for the relative rate constants, because the lattice energy can mask the reactivity of the solid compounds. The quantitative results are also difficult to reproduce because they are dependent upon the particle sizes, homogenization, amongst other factors. Considering only the liquid aldehydes in Table 9, it is observed that the electron-withdrawing substituent *para*-CF_3_ (σ_p_ = 0.54) [47] is marginally more reactive the unsubstituted reference (σ_p_ = 0), $k_{\mathrm{unsub}}/k_{para-{\mathrm{CF}}_{3}}\approx 0.95$, while the electron-donating *para*-OMe (σ_p_ = −0.27) [47] group slightly decreases the rate constant, $k_{\mathrm{unsub}}/k_{para-\mathrm{OMe}}\approx 2$, with respect to the reference. This trend is consistent with a nucleophilic attack at the carbonyl group. However, considering the large differences in the Hammett sigma of these substituents and the experimental uncertainties of these relative rate constants, may be possible that the access to the allylating agent may be the determining step, which limits the kinetics and leveled the reactivity. Certainly, more experiments with different substrates and different allylation reactants are required for a more conclusive assertion regarding the reactivity and the reaction mechanism under mechanochemical conditions. Such a task is beyond the scope of this contribution, but a more detailed study is being planned.

Given the excellent results obtained with potassium allyltrifluoroborate, it would be an interesting perspective to investigate the reactivity of potassium (2-substituted-allyl)trifluoroborate allylating agents as well as to explore the diastereoselectivity with potassium (3-substituted allyl)trifluoro borates. A detailed mechanistic study is also relevant, because if both carbonyl compound and allylating species are coordinated to the same metal centre, employing chiral catalysts could lead to the enantioselective allylation of ketones.

## 4. Materials and Methods

The aldehydes, ketones, carboxylic acids and potassium allyltrifluoroborate were purchased from Sigma Aldrich Chemical Co. (São Paulo, SP, Brazil) or Alfa Aesar (São Paulo, SP, Brazil) and used as received. The reactions were monitored by thin-layer chromatography on 0.25 mm silica gel 60 plates (F_254_) (E. Merck, Darmstadt, Germany) and GC HP5890 Series II system (HP, Palo alto, CA, USA), equipped with a HP-1 25 m × 0.32 microns column).

The products were purified, when necessary, by column chromatographic using silica gel 60 (230–400 mesh). ^1^H- and ^13^C-NMR data were obtained on a Varian Unity plus 300 or Varian UNMRS 400 spectrometer (Varian, Palo Alto, CA, USA) in CDCl_3_ using the solvent residual peak as the internal reference.

The experiments were performed with the Mini-Bead Beater from BioSpec (Bartlesville, OK, USA) at 70 Hz using 2.0 mL polypropylene screwcap microvials and glass beads of 1 mm de diameter and density of 2.5 g·cm^−3^ and with the vibratory mill MM200 model from Retsch (Haan, Germany) at 25 Hz using 5 mL stainless steel grinding jars and 10 mm stainless steel balls or 2 mL disposable Eppendorf jars and 1 mm glass beads. When using the disposable Eppendorf jars it is necessary to use a PTFE adapter for 5 samples, allowing tone to use 10 samples at once.

NMR spectra and X-ray powder diffraction (XRPD) patterns are available at the Supplementary Materials.

### 4.1. General Procedure for Synthesis of the Europium Catalyst: EuFum and MandEu

EuFum [Eu_2_(Fum)_3_(H_2_O)_4_·3H_2_O]_n_. Fumaric acid (1.5 mmol), europium nitrate (1.0 mmol), sodium hydroxide (3mmol) and deionized water (5 mL) were added to a 12 mL Teflon-lined vessel, which was sealed and heated at 160 °C for 12 h. The reaction vessel was allowed to slowly cool at room temperature. The solid was filtrated, washed with water, ethanol and acetone. Compound EuFum was isolated in 65% yield determined by titration of the lanthanide ion with standard EDTA solution using xylenol orange as an indicator.

MandEu [Eu(Mand)_3_(H_2_O)_2_]. Mandelic acid (1.5 mmol), europium oxide (0.5 mmol) and deionized water (5 mL) were stirred at room temperature for 4h. The solid was filtrated, washed with water, ethanol. Compound EuMand was isolated in 84% yield determined by titration of the lanthanide ion with standard EDTA solution using xylenol orange as an indicator.

### 4.2. General Procedure for the Allylation of Aldehydes with Potassium Allyltrifluoroborate *(**1**)* Using the Mini-Bead Beater

Potassium allyltrifluoroborate (0.11 mmol) and 20 glass beads (1 mm) were added to 2.0 mL polypropylene screwcap microvials and 100 μL of water. The reaction mixture was shaken at 70 Hz for 10 min. Aldehyde (0.10 mmol) was added and the mixture shaken for 5 to 10 min. The reaction mixture was extracted with dichloromethane (5 mL), the organic phase was dried over anhydrous magnesium sulfate. The solvent was removed under vacuum to yield compounds **3a**–**k**.

### 4.3. General Procedure for the Allylation of Aldehydes with Potassium Allyltrifluoroborate *(**1**)* Using the Retsch MM200 Mill

Method 1: Potassium allyltrifluoroborate (0.11 mmol), ketone (0.1 mmol) and 100 mg glass beads (1 mm) and solvent (1–100 μL) were added to the 2.0 mL disposable Eppendorf jar. The reaction mixture was shaken at 25 Hz for 15 min to 3 h. Then silica gel (100 mg) was added and the mixture milled for 2 min. The solid was transfer to a fritted funnel and washed with ethyl acetate (3 × 5 mL) and water (1 × 5 mL). The reaction mixture was extracted, the organic phase was dried over anhydrous magnesium sulfate and the solvent was removed under vacuum to yield compounds **5a**–**5f**, **2n**–**p**. If chromatographic column of the product was needed, the solid was transferred directly to the top of the column without the extraction step.

Method 2: Potassium allyltrifluoroborate (0.55 mmol), aldehyde or ketone (0.5 mmol) and two stainless steel balls (10 mm) and solvent (50–250 μL) were added to a 5.0 mL stainless steel jar. The reaction mixture was shaken at 25 Hz for 10 min to 3 h. Then silica gel (100 mg) was added and the mixture milled for 2 min. The solid was transfer to a fritted funnel and washed with ethyl acetate (3 × 5 mL) and water (1 × 5 mL). The reaction mixture was extracted with dichloromethane or ethyl acetate, the organic phase was dried over anhydrous magnesium sulfate. The solvent was removed under vacuum to yield compounds **3a**, **3c**–**e**, **3h**, **3l**–**m** and **5a**–**f**. If chromatographic column of the product was needed, the solid was transferred directly to the top of the column without the extraction step.

### 4.4. General Procedure for the Allylation of Acetophenone with Potassium Allyltrifluoroborate *(**1**)* Using EuFum or MandEu Catalysts in Solution

To a solution of the acetophenone (0.1 mmol) and the catalyst (10 mol %) in 1.1 mL of CH_2_Cl_2_:H_2_O (1:0.1) was added potassium allyltrifluoroborate (0.11 mmol). The biphasic mixture was stirred for the time indicated in Table 6. The reaction mixture was extracted with CH_2_Cl_2_ (3 × 5 mL) and dried over anhydrous magnesium sulfate. The solvent was removed in vacuum to yield compound **5a**.

*1-(4-Nitrophenyl)but-3-en-1-ol* (**3a**): ^1^H-NMR (400 MHz, CDCl_3_) δ 8.20 (d, *J* = 8.0 Hz, 2H), 7.53 (d, *J* = 8.0 Hz, 2H), 5.95–5.62 (m, 1H), 5.27–5.10 (m, 2H), 4.87 (dd, *J* = 8.0, 4,7 Hz, 1H), 2.70–2.38 (m, 2H), 2.28 (br. s, 1H). ^13^C-NMR (75.5 MHz, CDCl_3_) δ 151.0, 147.2, 133.2, 126.5, 123.6, 119.6, 72.1, 43.8 [23].

*1-(3-Nitrophenyl)but-3-en-1-ol* (**3b**): ^1^H-NMR (300 MHz, CDCl_3_) δ8.25 (s, 1H), 8.18–8.09 (m, 1H), 7.71 (d, *J* = 7.6 Hz, 1H), 7.53 (t, *J* = 7.9 Hz, 1H), 7.26 (s, 1H), 5.80 (m, 1H), 5.26–5.13 (m, 2H), 4.87 (dd, *J* = 7.9, 4.7 Hz, 1H), 2.66–2.41 (m, 2H), 2.25 (br. s, 1H). ^13^C-NMR (75.5 MHz, CDCl_3_) δ 148.4, 145.9, 133.2, 131.9, 129.3, 122.5, 120.8, 119.7, 72.0, 43.9 [25].

*1-(2-Nitrophenyl)but-3-en-1-ol* (**3c**): ^1^H-NMR (300 MHz, CDCl_3_) δ 7.93 (dd, *J* = 7.9, 1.5 Hz, 1H), 7.83 (d, *J* = 7.6 Hz, 1H), 7.65 (t, *J* = 7.6 Hz, 1H), 7.48–7.36 (m, 1H), 6.00–5.78 (m, 1H), 5.37–5.27 (m, 1H), 5.27–5.11 (m, 2H), 2.71 (ddd, *J* = 14.4, 6.75, 2.9 Hz, 1H), 2.51–2.32 (m, 2H). ^13^C-NMR (75 MHz, CDCl_3_) δ 147.7, 139.2, 133.9, 133.4, 128.1, 128.0, 124.3, 119.0, 68.3, 42.8 [48].

*1-(4-Chlorophenyl)but-3-en-1-ol* (**3d**):^1^H-NMR (300 MHz, CDCl_3_) δ 7.35–7.25 (m, 4H), 5.87–5.65 (m, 1H), 5.20–5.15 (m, 2H), 5.13 (d, *J* = 0.6 Hz, 1H), 4.71 (dd, *J* = 7.5, 5.3 Hz, 1H), 2.54–2.37 (m, 2H), 2.19 (br. s, 1H). ^13^C-NMR (75 MHz, CDCl_3_) δ 141.8, 133.5, 133.1, 128.1, 126.7, 118.4, 72.1, 43.4 [29].

*1-(4-Bromophenyl)but-3-en-1-ol* (**3e**): ^1^H-NMR (300 MHz, CDCl_3_) δ 7.47 (d, *J* = 8.2 Hz, 2H), 7.24 (d, *J* = 8.2 Hz, 2H), 5.90–5.64 (m, 1H), 5.27–5.05 (m, 2H), 4.80–4.57 (m, 1H), 2.65–2.31 (m, 2H), 2.13 (br. s., 1H). ^13^C-NMR (75 MHz, CDCl_3_) δ 142.8, 133.9, 131.4, 127.5, 121.2, 118.9, 72.6, 43.8 [29].

*1-(2-Methoxyphenyl)but-3-en-1-ol* (**3f**): ^1^H-NMR (300 MHz, CDCl_3_) δ 7.35 (dd, *J* = 7.6, 1.7Hz, 1H), 7.30–7.20 (m, 1H), 7.01–6.92 (m, 1H), 6.87 (d, *J* = 8.2, 1H), 5.97–5.76 (m,1H), 5.21–5.06 (m, 2H), 4.97 (dd, *J* = 7.9 Hz, 5.0 Hz, 1H), 3.85 (s, 3H), 2.68–2.43 (m, 3H). ^13^C-NMR (75 MHz, CDCl_3_) δ 156.2, 135.1, 131.7, 128.2, 126.7, 120.6, 117.4, 110.3, 6959, 55.1, 41.7 [49].

*1-(3-Methoxyphenyl)but-3-en-1-ol* (**3g**): ^1^H-NMR (300 MHz, CDCl_3_) δ 7.32–7.21 (m, 1H), 6.96–6.89 (m, 2H), 6.81 (dd, *J* = 8.0, 2.3 Hz, 1H), 5.81 (ddt, *J* = 17.2, 10.2, 7.1 Hz, 1H), 5.22–5.09 (m, 2H), 4.71 (dd, *J* = 7.4, 5.5 Hz, 1H), 3.81 (s, 3H), 2.49 (ddd, *J* = 12.5, 7.6, 4.5 Hz, 2H), 2.06 (s, 1H).^13^C-NMR (75 MHz, CDCl_3_) δ 159.7, 145.6, 134.4, 129.4, 118.5, 118.1, 112.9, 111.2, 73.2, 55.2, 43.8 [25].

*1-(4-Methoxyphenyl)but-3-en-1-ol* (**3h**): ^1^H-NMR (300 MHz, CDCl_3_) δ 7.30–7.23 (m, 2H), 6.90–6.85 (m, 2H), 5.78 (m, 1H), 5.18–5.06 (m, 2H), 4.66 (t, *J* = 6.5 Hz, 2H), 3.79 (s, 3H), 2.48 (t, *J* = 6.8 Hz, 1H).^13^C-NMR (75 MHz, CDCl_3_) δ 158.9, 136.0, 134.6, 127.0, 118.1, 113.7, 72.9, 55.2, 43.7 [16].

*1-(2-Methylphenyl)but-3-en-1-ol* (**3i**): ^1^H-NMR (400 MHz, CDCl_3_) δ 7.50–7.44 (m, 1H), 7.27–7.20 (m, 1H), 7.20–7.10 (m, 2H), 5.92–5.80 (m, 1H), 5.23–5.10 (m, 2H), 4.97 (m, 1H), 2.55–2.39 (m, 2H), 2.34 (s, 3H), 1.89 (s, 1H). ^13^C-NMR (100 MHz, CDCl_3_) δ 141.9, 134.7, 134.3, 130.3, 127.2, 126.2, 125.1, 118.2, 69.7, 42.6, 19.0 [50].

*1-(1-Naphthyl)but-3-en-1-ol* (**3j**): ^1^H-NMR (300 MHz, CDCl_3_) δ 8.13 (d, *J* = 8.2 Hz, 1H), 7.86 (d, *J* = 7.6 Hz, 1H), 7.72 (dd, *J* = 15.8, 7.6 Hz, 2H), 7.57–7.35 (m, 3H), 5.97 (m, 1H), 5.51 (m, 1H), 5.19–4.93 (m, 2H), 2.83–2.48 (m, 2H), 2.08 (d, *J* = 1.7 Hz, 1H). ^13^C-NMR (75 MHz, CDCl_3_) δ 139.8, 134.6, 132.9, 129.5, 127.8, 126.5, 124.8, 124.5, 124.4, 122.4, 122.1, 115.7, 69.1, 42.3 [50].

*1-(2-Naphthyl)but3-en-1-ol* (**3k**): ^1^H-NMR (300 MHz, CDCl_3_): δ 7.88–7.81 (m, 4H), 7.55–7.42 (m, 3H), 5.84 (m, 1H), 5.26–5.11 (m, 2H), 4.92 (m, 1H), 2.68–2.54 (m, 2H), 1.83 (br. s, 1H). ^13^C-NMR (75 MHz, CDCl_3_): δ 141.2, 134.3, 128.2; 127.9, 127.6, 126.1, 125.8, 118.5, 73.4, 43.7 [50].

*1-(3-Hydroxyphenyl)but-3-en-1-ol* (**3l**): ^1^H-NMR (300 MHz, CDCl_3_) δ 7.49–7.29 (m, 1H), 7.28–7.08 (m, 2H), 6.96–6.84 (m, 2H), 6.75 (dt, *J* = 8.4, 1.7 Hz, 1H), 5.80 (s, 1H), 5.23–5.09 (m, 2H), 5.01 (br. s, 1H), 4.71 (dd, *J* = 7.6, 5.3 Hz, 1H), 2.60–2.41 (m, 2H). ^13^C-NMR (75 MHz, CDCl_3_) δ: 155.7, 145.8, 134.3, 129.7, 118.6, 118.2, 114.5, 112.7, 73.0, 43.7 [51].

*1-(4-Hydroxyphenyl)but-3-en-1-ol* (**3m**): ^1^H-NMR (300 MHz, CDCl_3_) δ 7.25–7.19 (m, 2 H), 6.82-6.76 (m, 2 H), 5.89–5.62 (m, 1 H), 5.31 (br. s., 1H), 5.16–5.02 (m, 2 H), 4.68 (t, *J* = 6.5Hz, 1H), 2.62–2.39 (m, 2 H), 2.10 (br. s, 1 H). ^13^C-NMR (75 MHz, CDCl_3_) δ 155.1, 135.9, 134.5, 127.3, 118.4, 115.2, 73.0, 43.6 [52,53].

*1-Phenylbut-3-en-1-ol* (**3n**): ^1^H-NMR (300 MHz, CDCl_3_) δ 7.25–7.41 (m, 5H), 5.75–5.90 (m, 1H), 5.10–5.25 (m, 2H), 4.76 (t, 1H, *J* = 6.6 Hz), 2.45–2.60 (m, 2H). ^13^C-NMR (75 MHz, CDCl_3_) δ 43.7, 73.2, 118.3, 125.7, 127.5, 128.3, 134.4, 143.8 [48].

*1-(4-(Trifluoromethyl)phenyl)but-3-em-1-ol* (**3o**): ^1^H-NMR (400 MHz, CDCl_3_) δ: 7.61 (d, *J* = 8.2 Hz, 2H), 7,47 (d, *J* = 8.2Hz, 2H), 5.85–5.72 (m, 1H), 5.22–5.14 (m, 2H), 4.80 (dd, *J* = 8.0, 4.8 Hz, 1H), 2.50 (m, 2H), 2.06 (br. s, 1H).^13^C-NMR (100 MHz, CDCl_3_) δ: 147.7, 133.7, 129.7 (q, *J* = 32.7 Hz), 126.1, 125.3 (q, *J* = 3.8 Hz), 124.1(q, *J* = 270 Hz), 119.2, 72.5, 43.9 [54].

*Dec-1-en-4-ol* (**3p**): ^1^H-NMR (400 MHz, CDCl_3_) δ 5.96–5.75 (m, 1H), 5.22–5.03 (m, 2H), 3.65 (br. s, 1H), 2.51–2.00 (m, 2H), 1.52–1.18 (m, 10H), 0.89 (br. s, 3H). ^13^C-NMR (100 MHz, CDCl_3_) δ 134.9, 118.0, 70.7, 41.9, 36.8, 31.0, 29.3, 25.6, 22.6, 14.1 [16].

*1-Cyclohexylbut-3-en-1-ol* (**3q**): ^1^H-NMR (400 MHz, CDCl_3_) δ 5.84 (td, *J* = 16.4, 7.8 Hz, 1H), 5.24–4.88 (m, 2H), 3.40 (br. s, 1H), 2.32 (br. s, 1H), 2.18–2.00 (m, 1H), 1.96–1.57 (m, 6H), 1.52–0.96 (m, 6H). ^13^C-NMR (101 MHz, CDCl_3_) δ 135.4, 117.9, 74.7, 43.1, 38.8, 29.1, 28.1, 26.5, 26.3, 26.1 [48].

*2-Phenylpent-4-en-2-ol* (**5a**): ^1^H-NMR (300 MHz, CDCl_3_) δ 7.48–7.21 (m, 5H), 5.68–5.62 (m, 1H), 5.19–5.12 (m, 2H), 2.74–2.62 (dd, *J* = 12,0, 6.0 Hz, 1H), 2.54–2,46 (m, 1.0 Hz, 1H), 1,89 (br. s, 1H), 1.55 (s, 3H). ^13^C-NMR (75 MHz, CDCl_3_) δ 147.6, 133.6, 128.1, 126.6, 124.7, 119.45, 73.6, 48.4, 29.9 [55].

*2-(4-Nitrophenyl)pent-4-en-2-ol* (**5b**): ^1^H-NMR (400 MHz, CDCl_3_) δ 8.20 (d, *J* = 8.3 Hz, 2H), 7.62 (d, *J* = 8.3 Hz, 2H), 5.59 (dt, *J* = 16.9, 7.8 Hz, 1H), 5.23–5.09 (m, 2H), 2.61 (ddd, *J* = 22.2, 13.8, 7.9 Hz, 1H), 2.16 (s, 1H), 1.58 (s, br., 1H), 1.26 (s, 3H). ^13^C-NMR (101 MHz, CDCl_3_) δ 154.9, 132.4, 125.9, 123.4, 120.6, 73.5, 48.6, 29.9 [56].

*4-(2-Hydroxypent-4-en-2-yl)-phenol* (**5c**): ^1^H-NMR (400 MHz, CDCl_3_) δ 9.11 (s, 1H), 7.23–6.79 (m, 4H), 5.82 (td, *J* = 17.2, 7.5 Hz, 1H), 5.31–5.13 (m, 2H), 2.85 (dd, *J* = 14.0, 6.9 Hz, 1H), 2.74 (s, 1H), 2.57 (dd, *J* = 13.9, 7.8 Hz, 1H), 1.65 (s, 3H). ^13^C-NMR (101 MHz, CDCl_3_) δ 156.0, 132.9, 129.1, 125.9, 120.6, 119.45, 117.7, 46.5, 28.3 [49].

*2-(2-Methoxyphenyl)pent-4-en-2-ol* (**5d**): ^1^H-NMR (400 MHz, CDCl_3_) δ 7.31 (d, *J* = 7.7 Hz, 1H), 7.28–7.21 (m, 3H), 6.99–6.89 (m, 2H), 5.64 (td, *J* = 17.0, 7.1 Hz, 1H), 5.11–4.92 (m, 2H), 3.97–3.80 (m, 3H), 2.81 (dd, *J* = 13.7, 6.9 Hz, 1H), 2.60 (dd, *J* = 15.2, 6.1 Hz, 1H), 1.58 (s, 3H). ^13^C-NMR (101 MHz, CDCl_3_) δ 156.6, 134.7, 134.52, 128.2, 126.8, 120.8, 117.7, 111.24, 74.2, 55.3, 46.6, 26.9 [56].

*2-(4-Methoxyphenyl)pent-4-en-2-ol* (**5e**): ^1^H-NMR (400 MHz, CDCl_3_) δ 7.36 (d, *J* = 8.3 Hz, 2H), 6.87 (d, *J* = 8.3 Hz, 2H), 5.63 (dt, *J* = 17.4, 7.4 Hz, 1H), 5.19–5.05 (m, 1H), 3.80 (s, 3H), 2.64 (dt, *J* = 35.9, 18.0 Hz, 1H), 2.48 (dd, *J* = 13.7, 8.2 Hz, 1H), 1.89 (s, 1H), 1.53 (s, 3H). ^13^C-NMR (100 MHz, CDCl_3_) δ 158.3, 139.8, 133.8, 125.9, 119.3, 113.4, 73.3, 55.2, 48.5, 29.9 [57].

*1-Allylcyclohexanol* (**5f**): ^1^H-NMR (400 MHz, CDCl_3_) δ 5.97–5.82 (m, 1H), 5.15 (m, 2H), 2.22 (d, *J* = 7.5 Hz, 2H), 1.72–1.37 (m, 10H), 1.35–1.21 (m, 1H). ^13^C-NMR (101 MHz, CDCl_3_) δ 133.7, 118.6, 70.9, 46.7, 37.4, 25.7, 22.2 [58].

*3-Methylhex-5-en-3-ol* (**5g**): (400 MHz, CDCl_3_) d 5.92–5.80 (m, 1H), 5.20–5.05 (m, 2H), 2.20 (d, *J* = 7.4 Hz, 2H), 1.51 (q, *J* = 7.4 Hz, 2H), 1.16 (s, 3H), 0.92 (t, *J* = 7.4 Hz, 3H); ^13^C-NMR (100 MHz, CDCl_3_) d 134.1, 118.5, 72.3, 45.8, 34.2, 26.1, 8.1 [59].

## 5. Conclusions

A mechanochemical method was successfully developed for the allylation of carbonyl compounds by potassium allyltrifluoroborate. By controlling the mechanical energy, the method can be made specific to allylation of aldehydes in the presence of ketones at short reaction times. Whereas the allylation of ketones was shown, for the first time, to be effective by mechanochemistry with larger mechanical energy, which can be made practically quantitative by using lanthanide catalysts. The most efficient procedures employed water as liquid assisted grinding (LAG) or as solvent and because of the allylation agent (allyltrifluoroborate) and its by-products are inert, nontoxic, water soluble, readily available salts and can be easily removed from the reaction medium, the methodologies developed conform with most of the green chemistry principles.

## Figures and Tables

**Table 1:** Mechanochemical allylation of *p*-nitrobenzaldehyde (**2a**) by potassium allyltrifluoroborate (**1**): solvent effects ^1^.

Experiment	Solvent ^2^	Yield (%) ^3^
1	none	0
2	CH_2_Cl_2_	23 ^4^
3	CH_2_Cl_2_:H_2_O (99:1)	54
4	CH_2_Cl_2_:H_2_O (8:2)	78
5	CH_2_Cl_2_:H_2_O (1:1)	90
6	H_2_O	97
7 ^5^	H_2_O	99

^1^ Milling (BioSpec) by 20 min at 70 Hz, room temperature; ^2^ 100 μL; ^3^ Isolated yield; ^4^ Conversion yield by GC; ^5^ One pot-two steps procedure: 10 min milling of potassium allyltrifluoroborate and water followed by the addition of *p*-nitrobenzaldehyde and 10 min milling.

**Table 2:** Mechanochemical allylation of arylaldehydes by potassium allyltrifluoroborate (**1**): substituent effects ^1^.

Entry	Substrate	Aryl	Yield (%) ^2^
1	**2a**	4-NO_2_-C_6_H_4_	99
2	**2b**	2-NO_2_-C_6_H_4_	94
3	**2c**	3-NO_2_-C_6_H_4_	89
4	**2d**	4-Cl-C_6_H_4_	94
5	**2e**	4-Br-C_6_H_4_	97
6	**2f**	2-MeO-C_6_H_4_^3^	53
7	**2g**	3-MeO-C_6_H_4_^3^	82
8	**2h**	4-MeO-C_6_H_4_^3^	78
9	**2i**	2-Me-C_6_H_4_^3^	62
10	**2j**	1-naftyl ^3^	62 ^4^
11	**2k**	2-naftyl	85

^1^ Potassium allyltrifluoroborate (**1**) and 100 μL of water are milled (BioSpec, 70 Hz, room temperature) for 10 min followed by the addition of the arylaldehyde and 10 min milling; ^2^ isolated yield; ^3^ the substrate is a liquid; ^4^ conversion yield by GC.

**Table 3:** Mechanochemical allylation of selected substituted benzaldehyde by potassium allyltrifluoroborate (**1**) ^1^.

Entry	Substrate	Substituent	Yield (%) ^2^
1	**2a**	4-NO_2_	87
2	**2c**	3-NO_2_	92
3	**2d**	4-Cl	72
4	**2e**	4-Br	82
5	**2h**	4-MeO ^3^	60 ^4^
6	**2l**	3-HO	95
7	**2m**	4-HO	73
8	**2a**	4-NO_2_^5^	25
9	**2l**	3-HO ^3,6^	41
10	**2h**	4-MeO ^3,5^	14
11	**2h**	4-MeO ^3,6^	3
12	**2a**	4-NO_2_^7^	0
13	**2a**	4-NO_2_^8^	78

^1^ Potassium allyltrifluoroborate, substituted benzaldehyde, and 250 μL of water are milled for 20 min in a Retsch MM200 at 25 Hz, with stainless steel 5 mL vial, two 10 mm stainless steel balls, and room temperature; ^2^ isolated yield; ^3^ Substrate is a liquid; ^4^ Conversion yield by GC; ^5^ Grinding auxiliary SiO_2_(s); ^6^ Grinding auxiliary NaCl (s); ^7^ Without water upon 1 h of milling; ^8^ Using 50 μL of water upon 30 min of milling.

**Table 4:** Mechanochemical allylation of acetophenone (**3a**) by potassium allyltrifluoroborate (**1**) ^1^.

Entry	LAG	Catalyst	Yield (%) ^2^
1	-	-	0
2	CH_2_Cl_2_:H_2_O (7 µL:1 µL)	-	2
3	H_2_O (7 µL)	-	9
4	H_2_O (100 µL)	-	17
5	-	MandEu	27
6	H_2_O (7 µL)	MandEu	70
7	H_2_O (100 µL)	MandEu	72
8	CH_2_Cl_2_:H_2_O (7 µL:1 µL)	MandEu	79

^1^ Vibratory mill Retsch MM200 at 25 Hz, potassium allyltrifluoroborate (0.11 mmol), acetophenone (0.10 mmol), 10 mol % MandEu (when present), 2.0 mL Eppendorf jar, 100 mg of 1 mm glass beads, milling for 3 h; ^2^ Conversion yield by GC.

**Table 5:** Mechanochemical allylation of acetophenone (**3a**) by potassium allyltrifluoroborate (**1**) ^1^.

Entry	Addition of 1	Catalyst	Reaction Time (h)	Yield (%) ^2^
1	1 × (0.55 mmol) at starting	-	0.5	14
2	1 × (0.55 mmol) at starting	-	3	40
3	1 × (0.2 mmol) at starting	-	0.5	4
4	3 × (0.2 mmol) in 30 min intervals	-	3	87 ^3^
5	1 × (0.55 mmol) at starting	MandEu	3	54
6	1 × (0.2 mmol) at starting	MandEu	0.5	34
7	3 × (0.2 mmol) in 30 min intervals	MandEu	3	100

^1^ Vibratory mill Retsch MM200 at 25 Hz, potassium allyltrifluoroborate (0.55 mmol), acetophenone (0.50 mmol), 50 µL H_2_O (LAG), 10 mol % MandEu (when present), 5 mL stainless steel jar, two 10 mm stainless steel balls; ^2^ Conversion yield by GC; ^3^ 13% of starting material.

**Table 6:** Allylation of acetophenone (**3a**) by potassium allyltrifluoroborate (**1**) in solution ^1^.

Entry	Solvent	Catalyst	Reaction time (h)	Yield (%) ^2^
1	CH_2_Cl_2_:H_2_O	-	3	15
2	CH_2_Cl_2_:H_2_O	EuFum	2	36
3	CH_2_Cl_2_:H_2_O	EuFum	24	46
4	CH_2_Cl_2_:H_2_O	MandEu	2	63
5	CH_2_Cl_2_:H_2_O	MandEu	3	73
6	H_2_O	MandEu	3	24

^1^ Potassium allyltrifluoroborate (0.11 mmol), acetophenone (0.10 mmol) and 10 mol % of catalyst, in CH_2_Cl_2_:H_2_O (1 mL:0.1 mL) or H_2_O (1 mL) under constant stirring at room temperature; ^2^ Conversion yield by GC.

**Table 7:** Catalyzed mechanochemical allylation of substituted acetophenones (**4a**–**e**) by potassium allyltrifluoroborate (**1**) ^1^.

Entry	Substrate	Substituent	Yield (%) ^2^
1	**4a**	H	100
2	**4b**	4-NO_2_	62 ^3^
3	**4c**	4-OH	74 ^4^
4	**4d**	2-OMe	63 ^5^
5	**4e**	4-OMe	41 ^6^

^1^ Vibratory mill Retsch MM200 at 25 Hz, potassium allyltrifluoroborate addition in three 0.2 mmol portions at 30 min intervals, substituted acetophenone (0.50 mmol), 10 mol % MandEu, 50 µL H_2_O (LAG), 5 mL stainless steel jar, two 10 mm stainless steel balls, milling for 3 h; ^2^ Conversion by GC; ^3^ 17% of starting material and 21% of by-products; ^4^ 26% of by-products; ^5^ 37% of starting material; ^6^ 59% of starting material.

**Table 8:** Mechanochemical allylation of aliphatic aldehydes, aliphatic ketones, and benzaldehyde by potassium allyltrifluoroborate (**1**) ^1^.

Entry	Substrate		Catalyst	Milling	Yield (%) ^2^
1	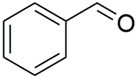	(**2n**)	-	0.5 h	96
2	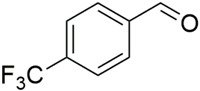	(**2o**)	-	0.5 h	95
3	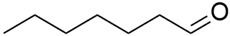	(**2p**)	-	0.5 h	96
4	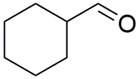	(**2q**)	-	0.5 h	99
5	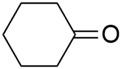	(**4f**)	MandEu	3 h	96
6	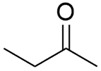	(**4g**)	MandEu	3 h	76 ^3^

^1^ Vibratory mill Retsch MM200 at 25 Hz, potassium allyltrifluoroborate (0.11 mmol), substrate (0.10 mmol), 7 µL H_2_O (LAG), 10 mol % MandEu (when present), 2.0 mL Eppendorf jar, 100 mg of 1 mm glass beads; ^2^ Conversion yield by GC; ^3^ Conversion yield by ^1^H-NMR.

**Table 9:** Competitive mechanochemical allylation of substituted (**2a**, **2h**, **2o** and **4b**, **4e**) and unsubstituted aromatic carbonyl compounds (**2n** and **4a**) by potassium allyltrifluoroborate (**1**).

Entry	Substrates	Substituents	Ratio ^3^
1 ^1^	**2n**/**2a**	H/4-NO_2_	14.5; 4.3
2 ^1^	**2n**/**2h**	H/4-OMe	2.2; 2.0
3 ^2^	**2n**/**2o**	H/4-CF_3_	0.96; 0.95
4 ^2^	**4a**/**4b**	H/4-NO_2_	1.7; 2.4
5 ^2^	**4a**/**4e**	H/4-OMe	2.4; 2.1

^1^ Vibratory mill Retsch MM200 at 25 Hz, substituted benzaldehyde (0.50 mmol), benzaldehyde (0.50 mmol), potassium allyltrifluoroborate (0.1 mmol), 7 µL H_2_O (LAG), 2.0 mL Eppendorf jar, 20 glass beads (1 mm), milling for 30 min; ^2^ Vibratory mill Retsch MM200 at 25 Hz, substituted acetophenone (0.50 mmol), acetophenone (0.50 mmol), potassium allyltrifluoroborate (0.1 mmol), 10 mol % MandEu, 7 µL H_2_O (LAG), 2.0 mL Eppendorf jar, 100 mg of 1 mm glass beads, milling for 3 h; ^3^ Ratio between the areas in the GC of unsubstituted and substituted products obtained from two distinct experiments.

## Supplementary Materials

The following are available online at http://www.mdpi.com/1420-3049/21/11/1539/s1, Figures S1–S50: NMR spectra and X-ray powder diffraction (XRPD) patterns.
