# Supplementary material for: A Green Approach for Allylations of Aldehydes and Ketones: Combining Allylborate, Mechanochemistry and Lanthanide Catalyst
Source: Molecules. 2016 Nov 16;21(11):1539. doi: 10.3390/molecules21111539 (PMC6273256; doi:10.3390/molecules21111539)
Supplement: Supplementary file 1 [file molecules-21-01539-s001.pdf]

Supplementary Materials: A Green Approach for Allylations of Aldehydes and Ketones: Combining Allylborate, Mechanochemistry and Lanthanide Catalyst

Viviane P. de Souza, Cristiane K. Oliveira, Thiago M. de Souza, Paulo H. Menezes, Severino Alves Jr., Ricardo L. Longo and Ivani Malvestiti

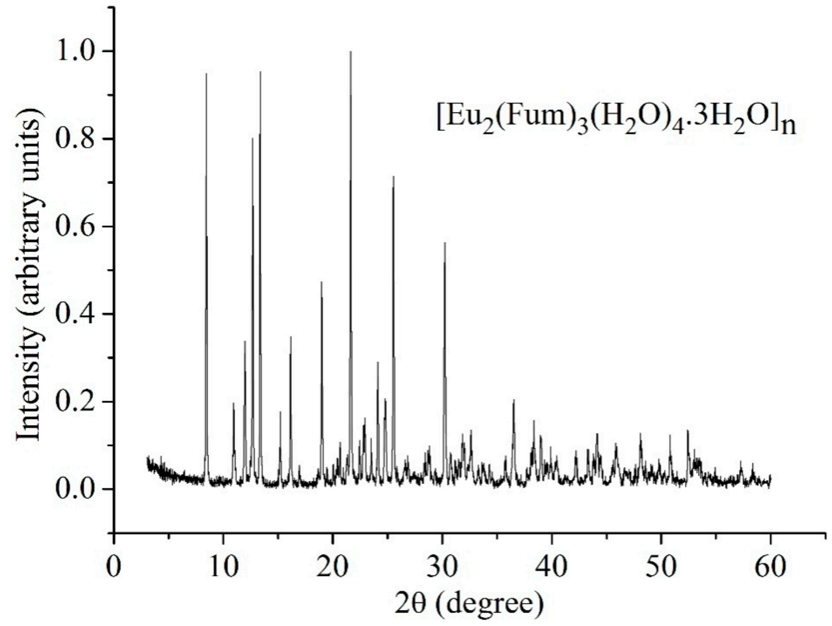

Figure S1. X-ray powder diffraction (XRPD) patterns for MOF-$[Eu_2(Fum)_3(H_2O)_4{\cdot}3H_2O]_n$: EuFum.

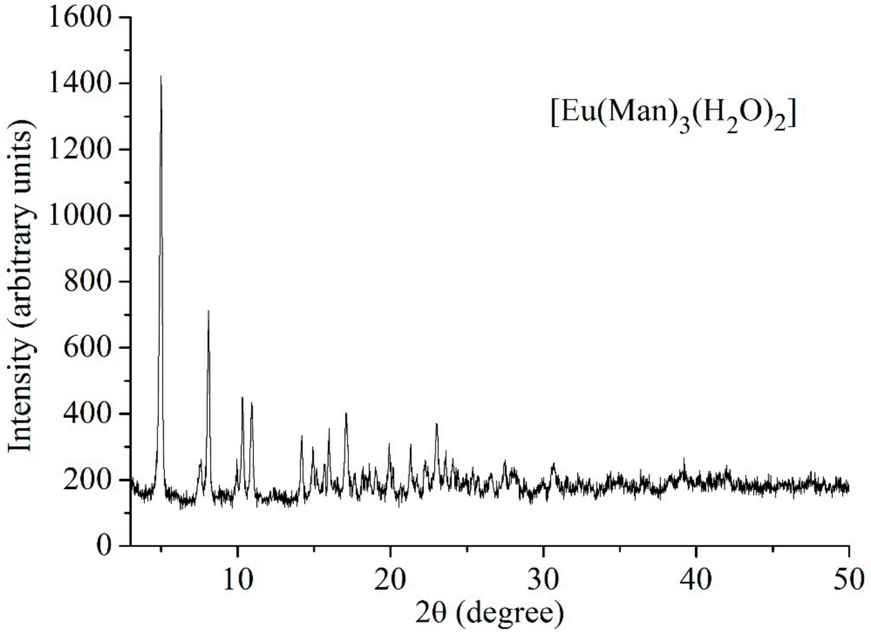

Figure S2. X-ray powder diffraction (XRPD) patterns for $[Eu_2(Man)_3(H_2O)_2]$: MandEu.

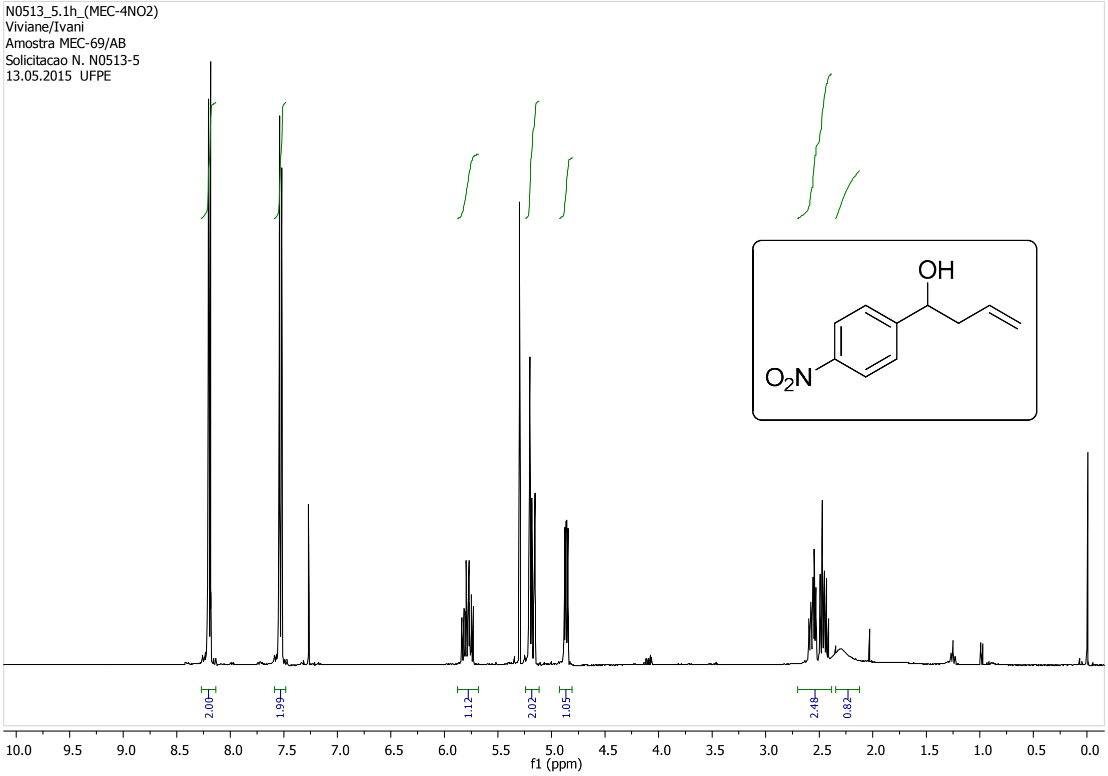

Figure S3. ^{1}H-NMR (300 MHz, $CDCl_3$) of 1-(4-Nitrophenyl)but-3-en-1-ol (**3a**).

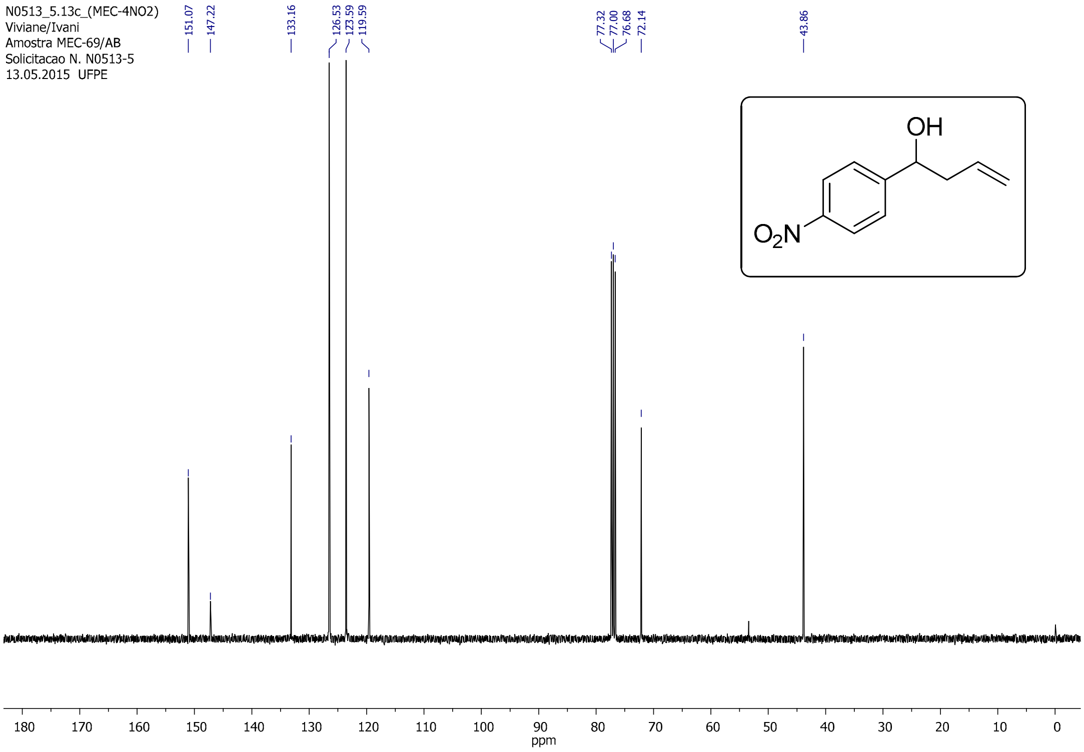

Figure S4. ^{13}C-NMR (75 MHz, $CDCl_3$) of 1-(4-Nitrophenyl)but-3-en-1-ol (**3a**).

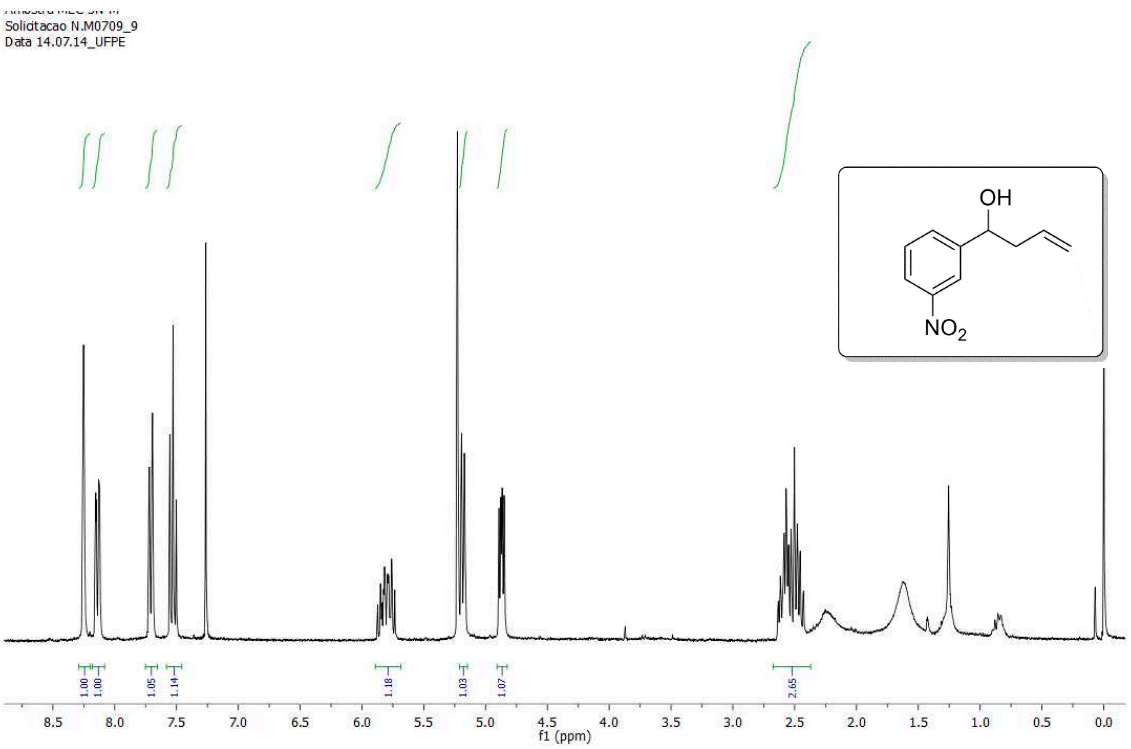

Figure S5. ^{1}H-NMR (300 MHz, $CDCl_3$) of 1-(3-Nitrophenyl)but-3-en-1-ol (**3b**).

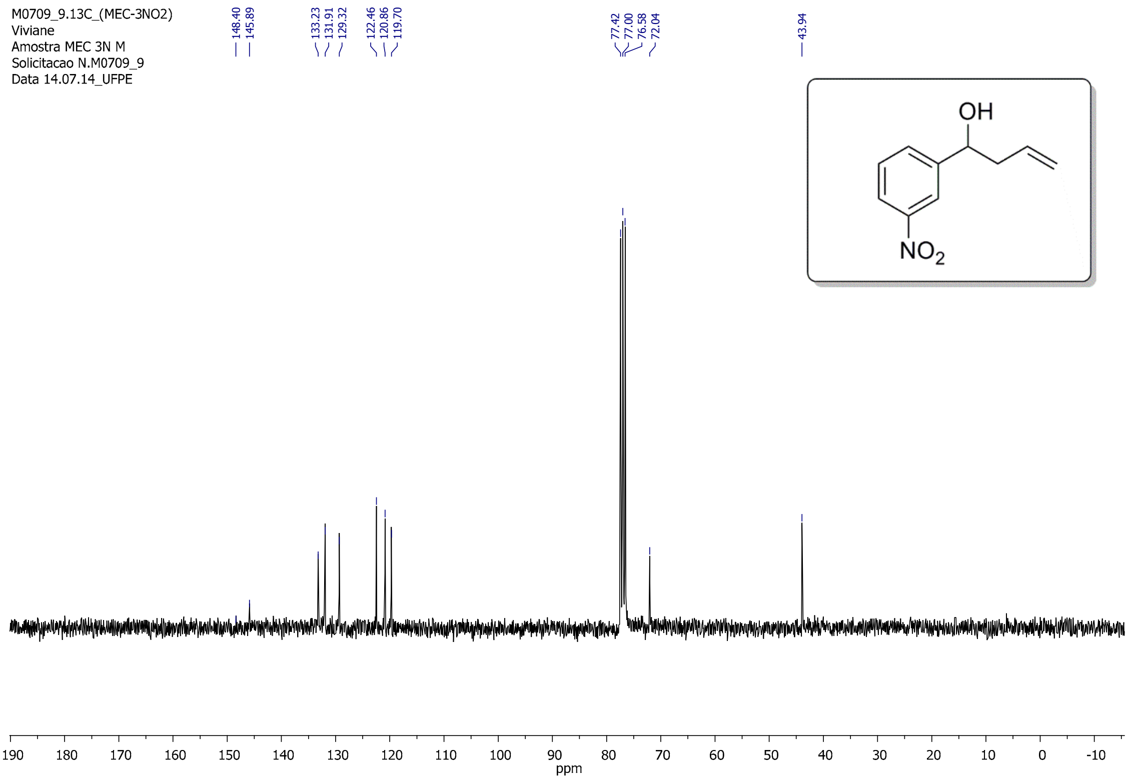

Figure S6. ^{13}C-NMR (75 MHz, $CDCl_3$) of 1-(3-Nitrophenyl)but-3-en-1-ol (**3b**).

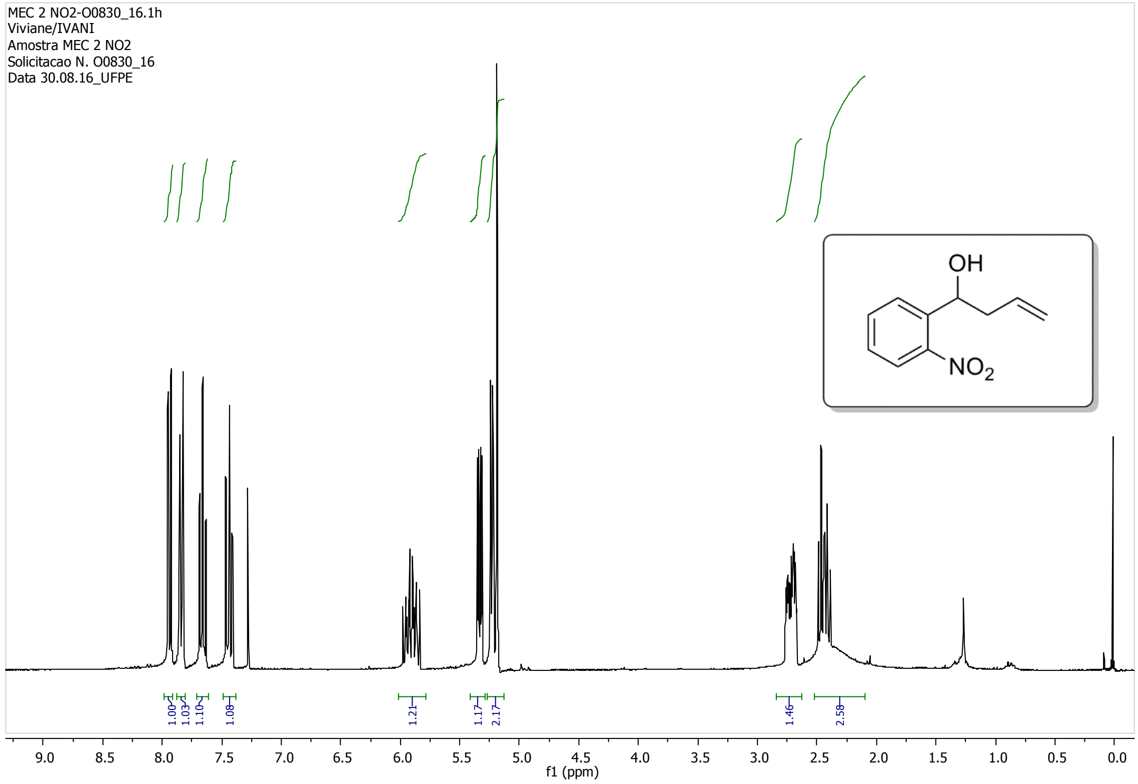

Figure S7. ^{1}H-NMR (300 MHz, $CDCl_3$) of 1-(2-Nitrophenyl)but-3-en-1-ol (**3c**).

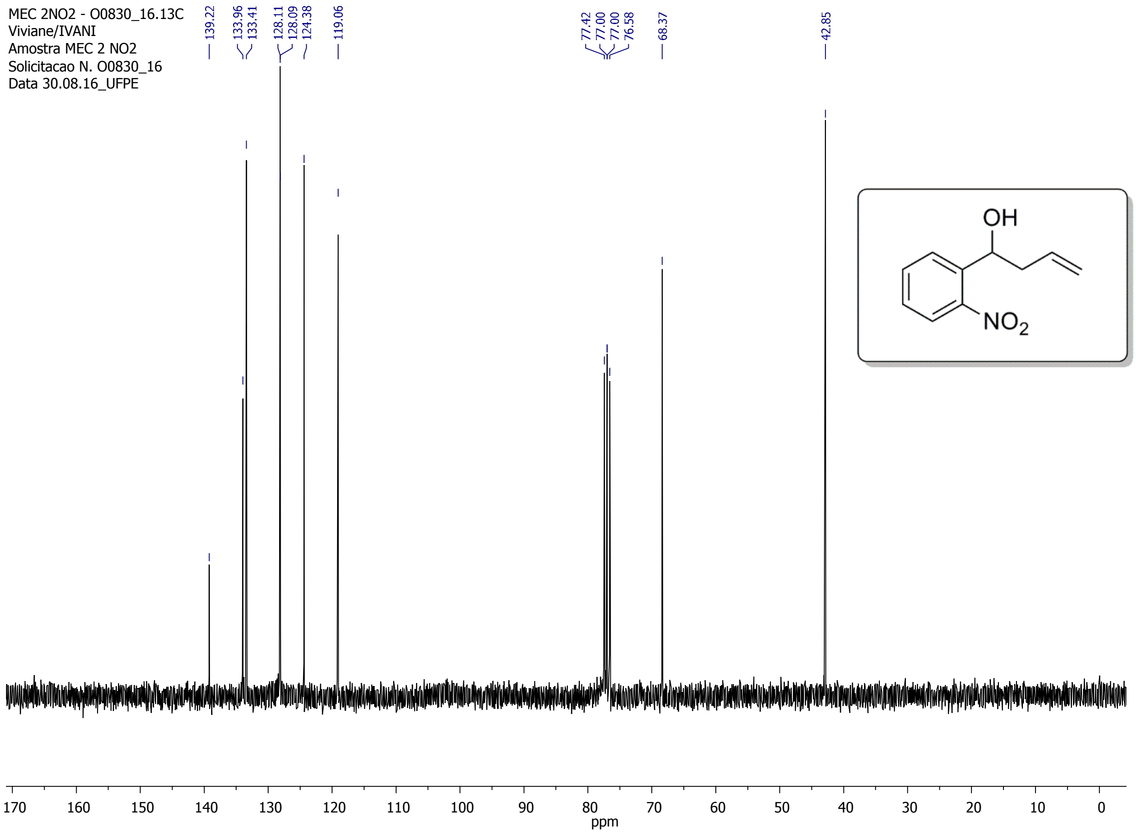

Figure S8. ^{13}C-NMR (75 MHz, $CDCl_3$) of 1-(2-Nitrophenyl)but-3-en-1-ol (**3c**).

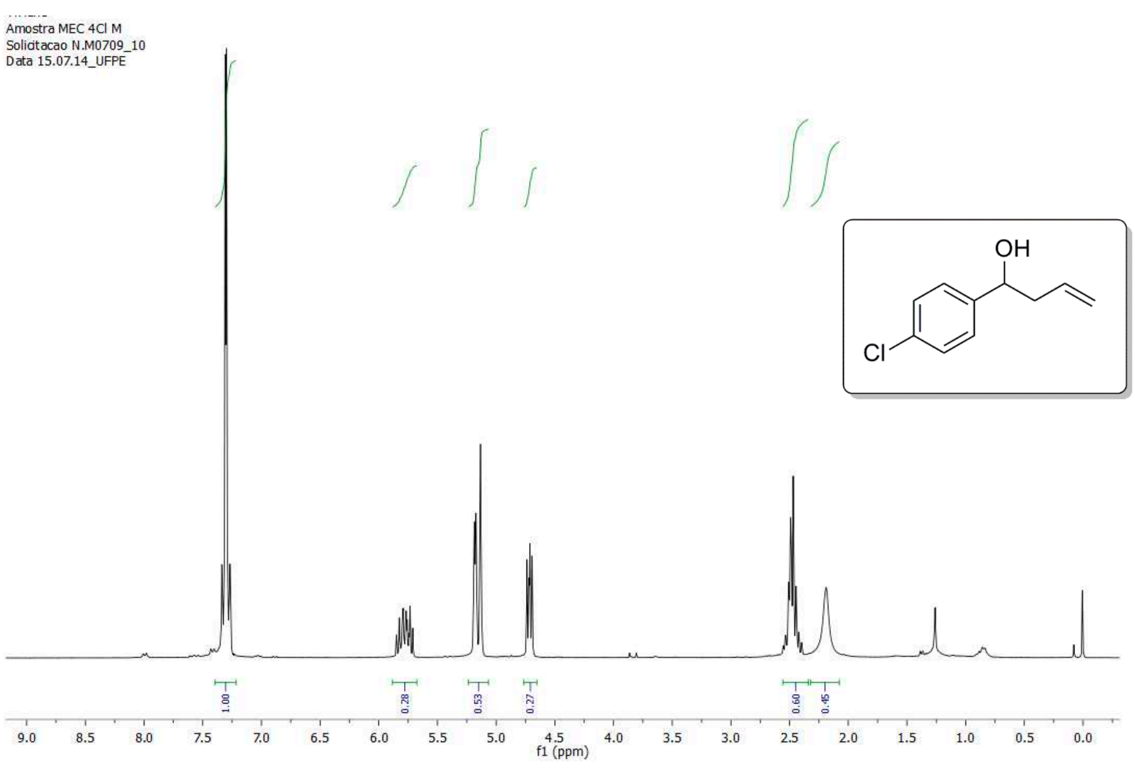

Figure S9. ^{1}H-NMR (300 MHz, $CDCl_3$) of 1-(4-chlorophenyl)but-3-en-1-ol (**3d**).

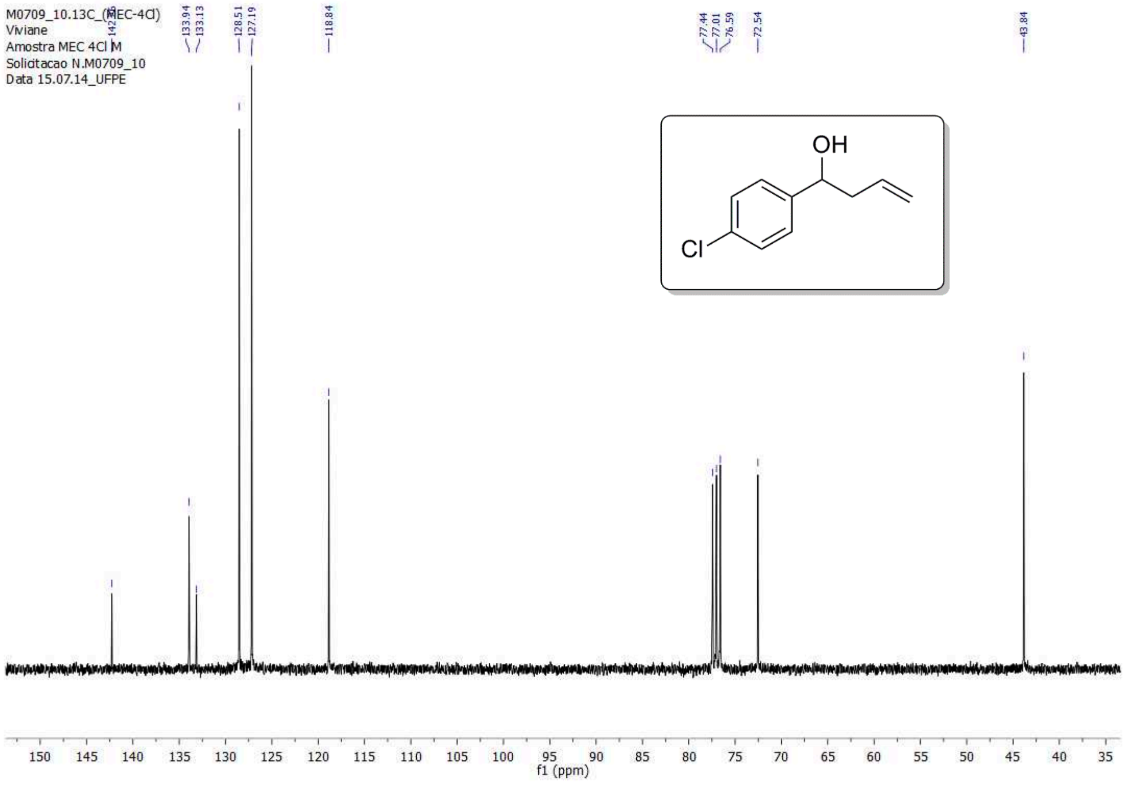

Figure S10. ^{13}C-NMR (75 MHz, $CDCl_3$) of 1-(4-chlorophenyl)but-3-en-1-ol (**3d**).

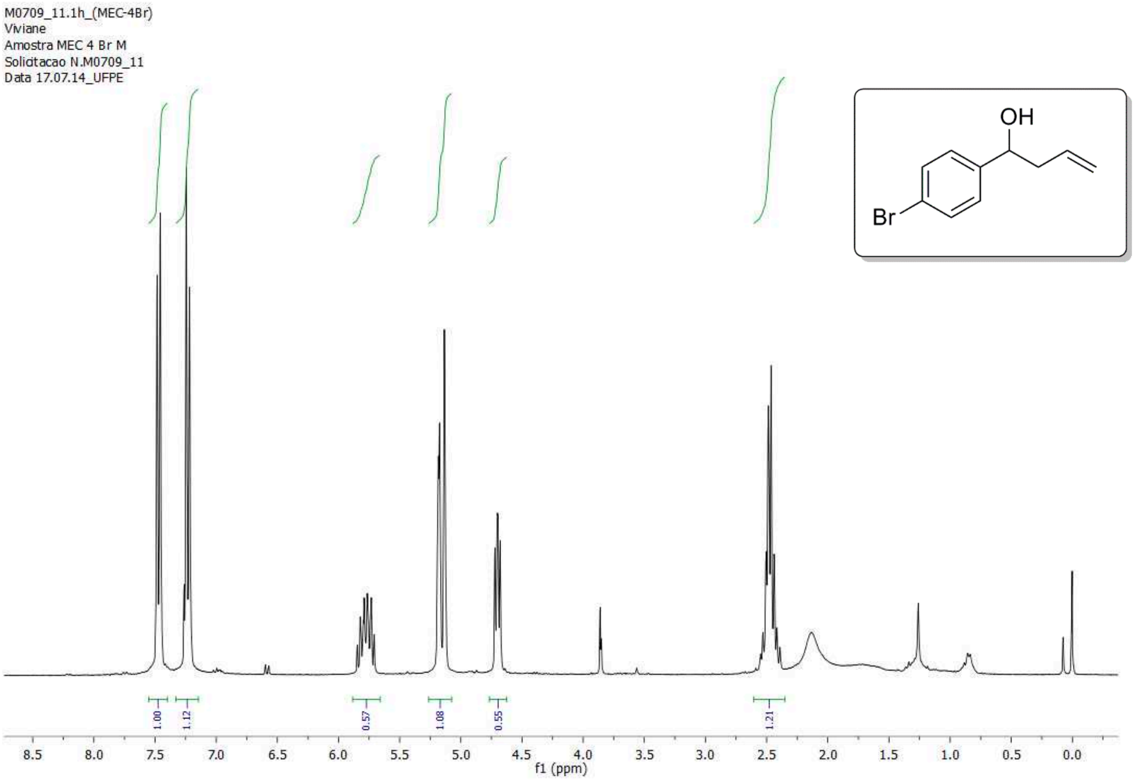

Figure S11. ^{1}H-NMR (300 MHz, $CDCl_3$) of1-(4-bromophenyl)but-3-en-1-ol (**3e**).

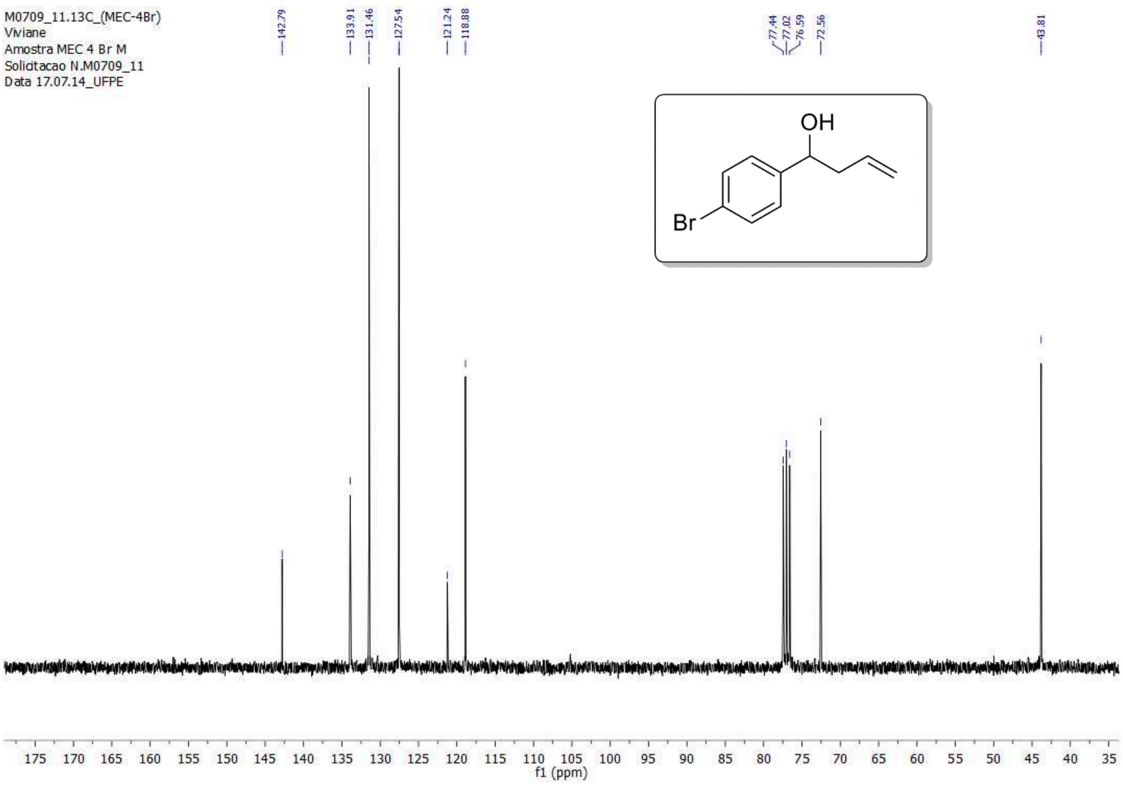

Figure S12. ^{13}C-NMR (75 MHz, $CDCl_3$) of 1-(4-bromophenyl)but-3-en-1-ol (**3e**).

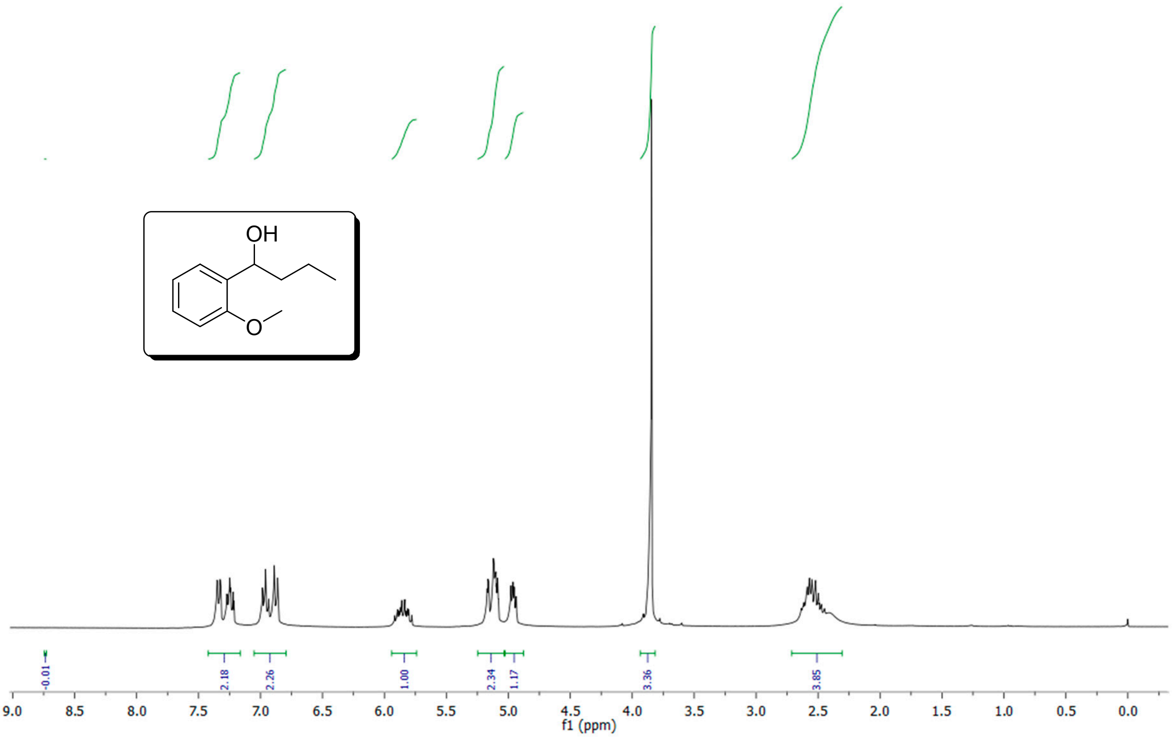

Figure S13. ^{1}H-NMR (300 MHz, $CDCl_3$) of 1-(2-Methoxyphenyl)but-3-en-1-ol (**3f**).

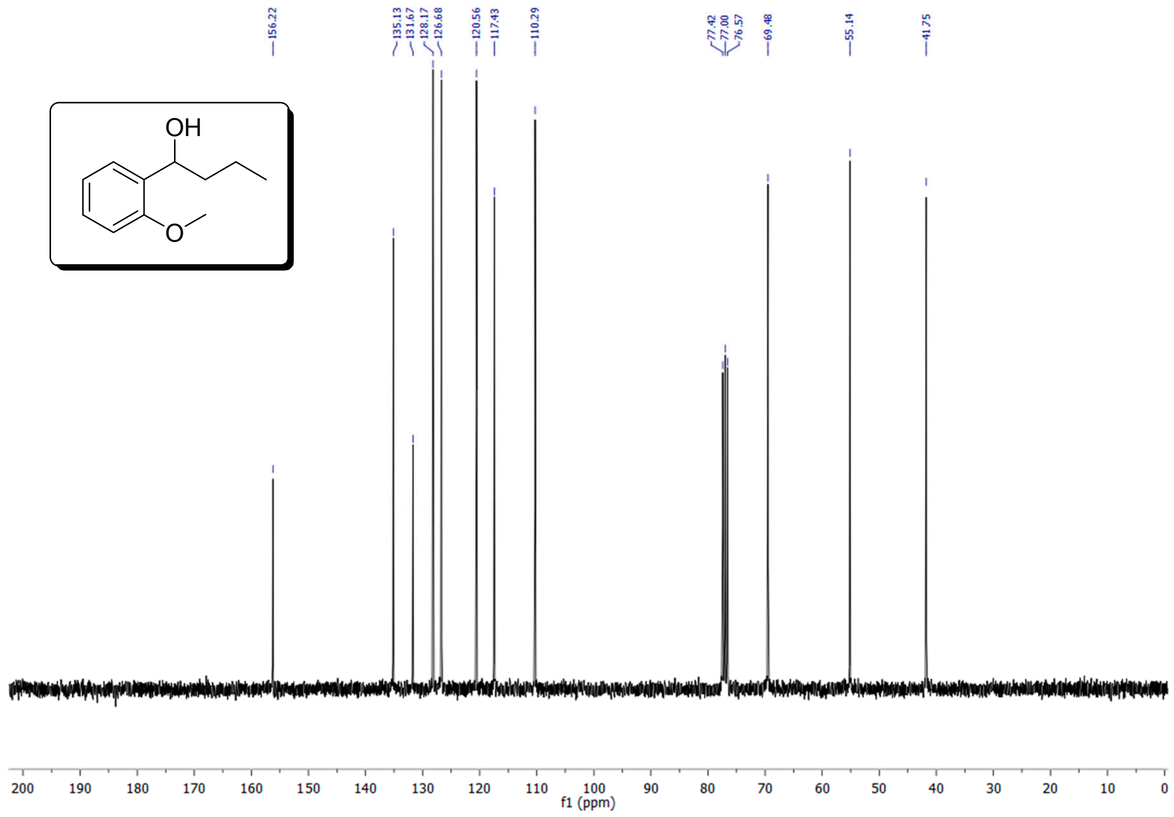

Figure S14. ^{13}C-NMR (75 MHz, $CDCl_3$) of 1-(2-Methoxyphenyl)but-3-en-1-ol (**3f**).

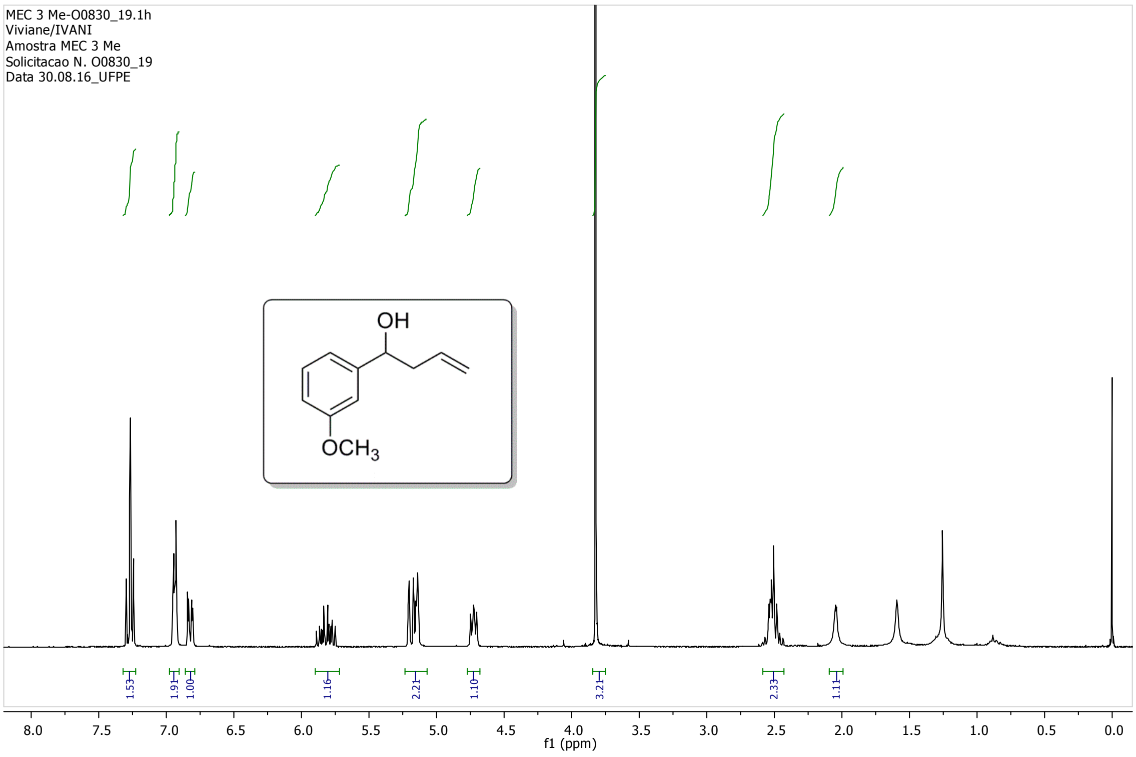

Figure S15. ^{1}H-NMR (300 MHz, $CDCl_3$) of 1-(3-Methoxyphenyl)but-3-en-1-ol (**3g**).

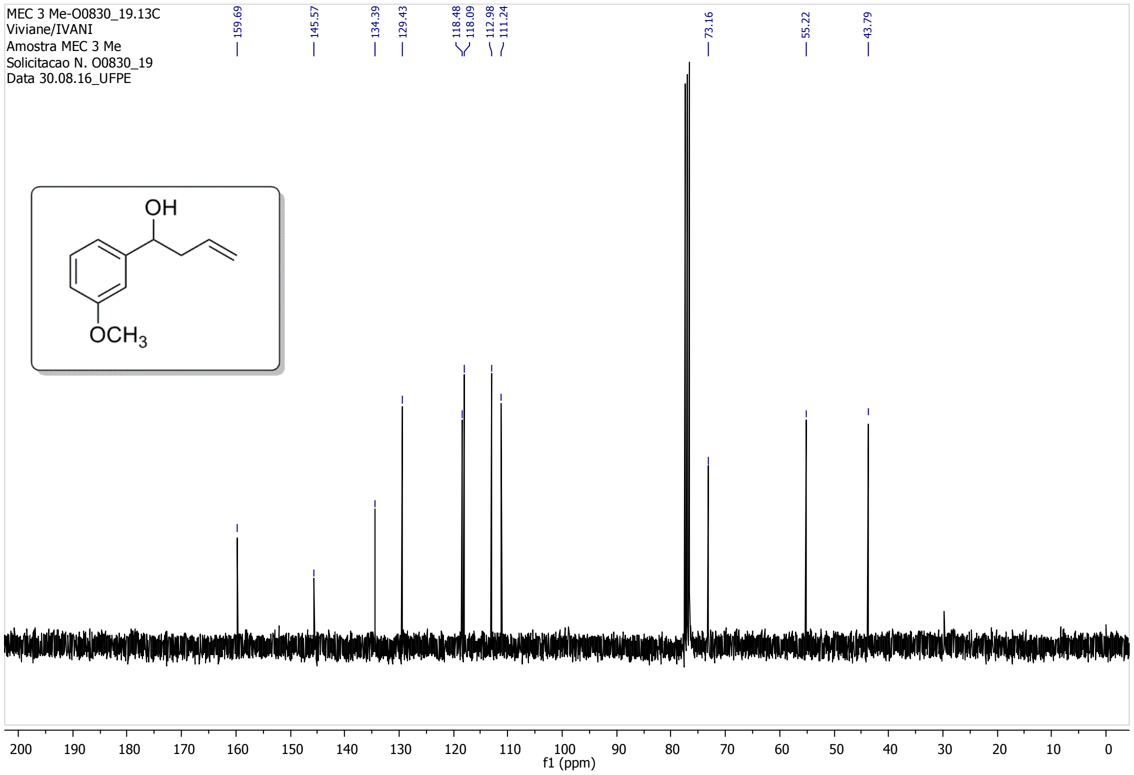

Figure S16. ^{13}C-NMR (75 MHz, $CDCl_3$) of 1-(3-Methoxyphenyl)but-3-en-1-ol (**3g**).

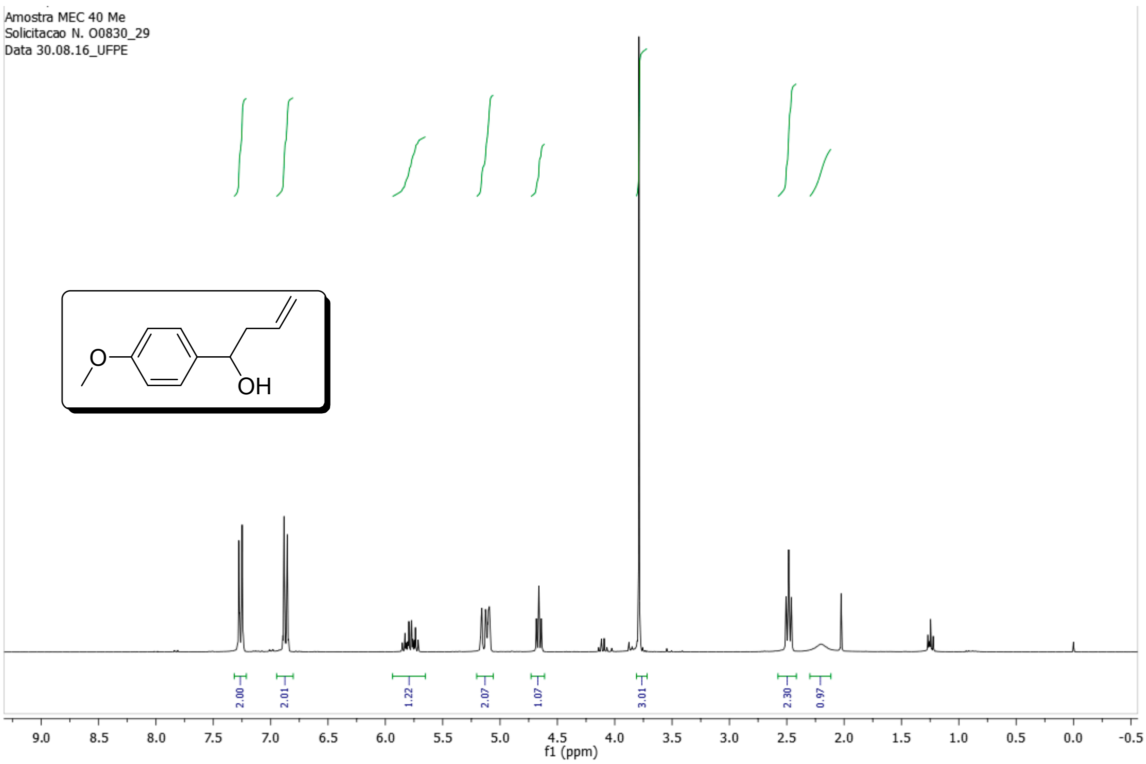

Figure S17. ^{1}H-NMR (300 MHz, $CDCl_3$) of 1-(4-Methoxyphenyl)but-3-en-1-ol (**3h**).

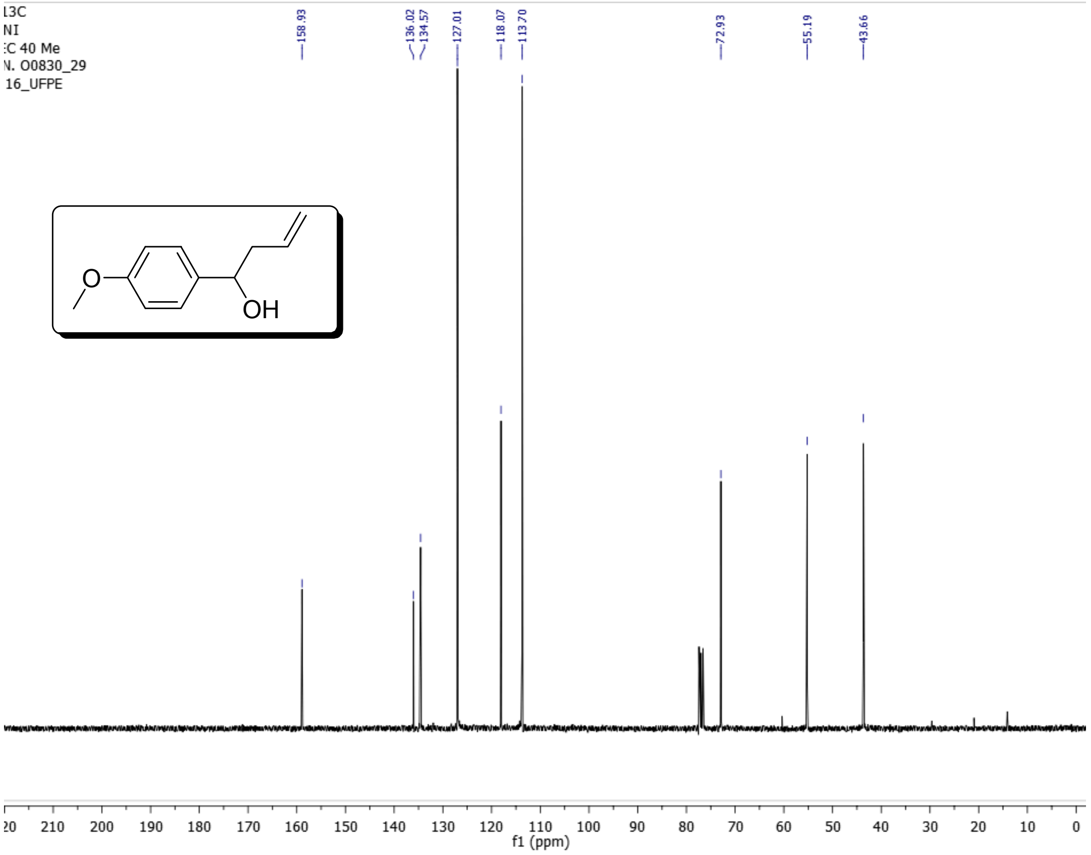

Figure S18. ^{13}C-NMR (75 MHz, $CDCl_3$) of 1-(4-Methoxyphenyl)but-3-en-1-ol (**3h**).

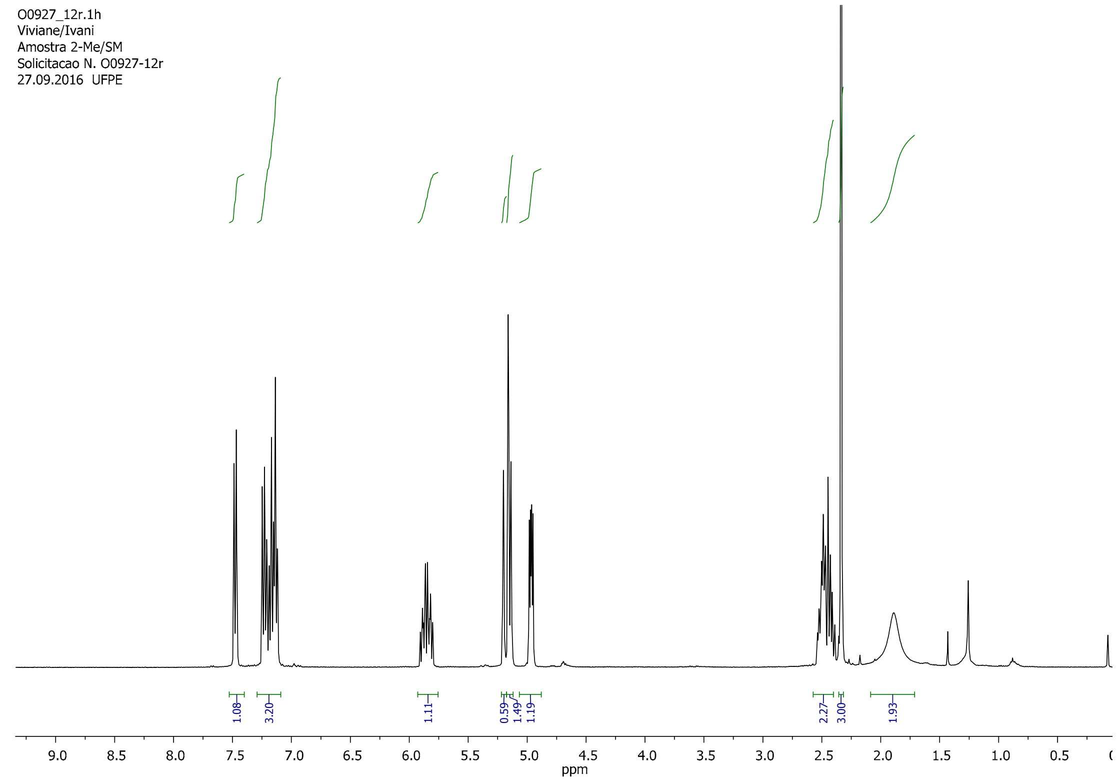

Figure S19. ^{1}H-NMR (300 MHz, $CDCl_3$) of 1-(2-Methylphenyl)but-3-en-1-ol (**3i**).

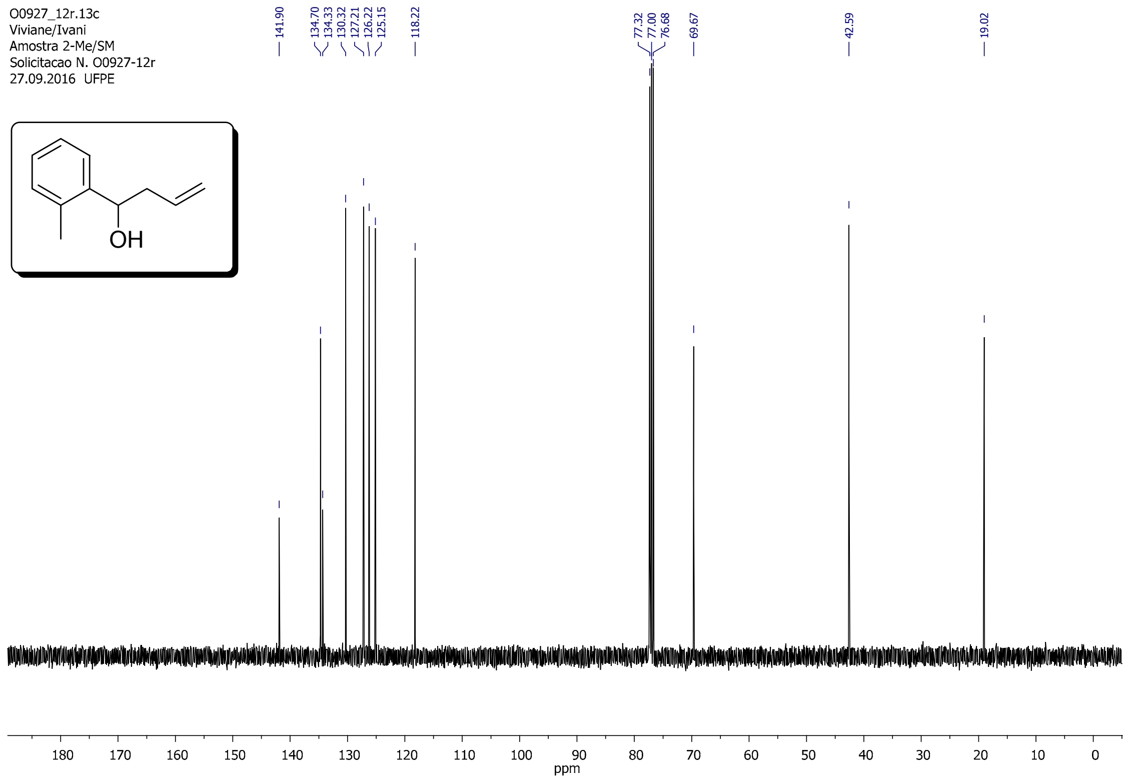

Figure S20. ^{13}C-NMR (75 MHz, $CDCl_3$) of 1-(2-Methylphenyl)but-3-en-1-ol (**3i**).

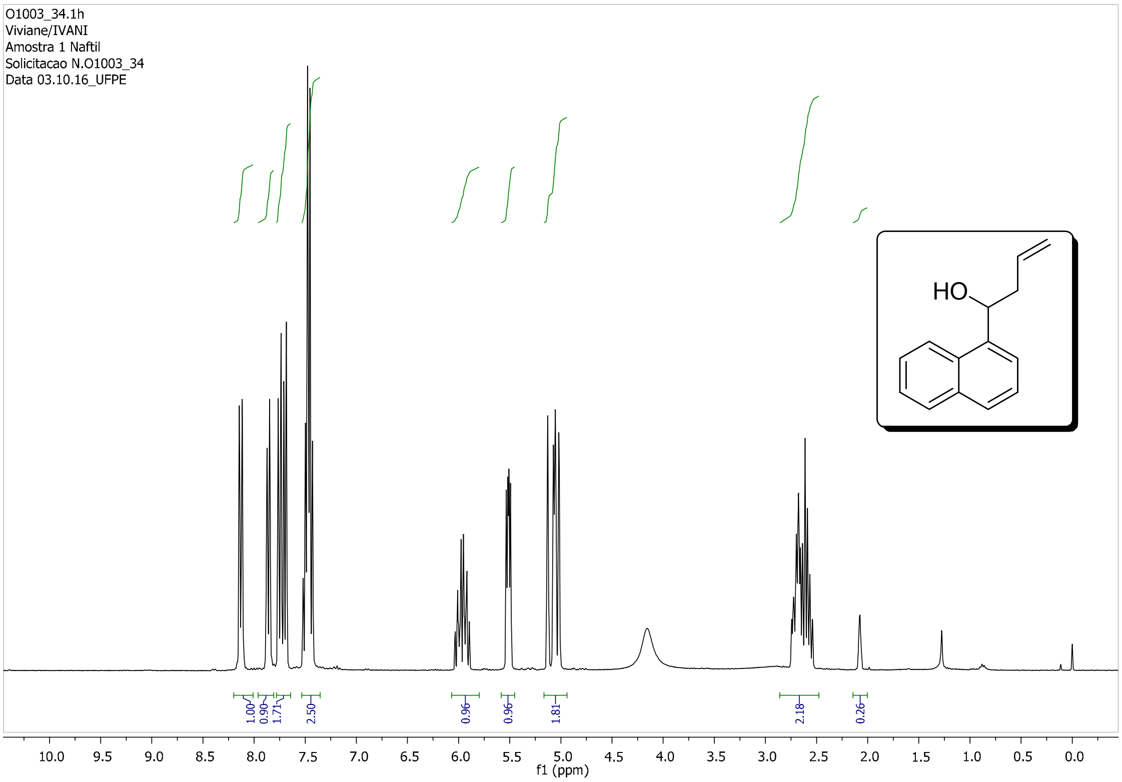

Figure S21. ^{1}H-NMR (300 MHz, $CDCl_3$) of 1-(naphtho-1-yl)but-3-en-1-ol (**3j**).

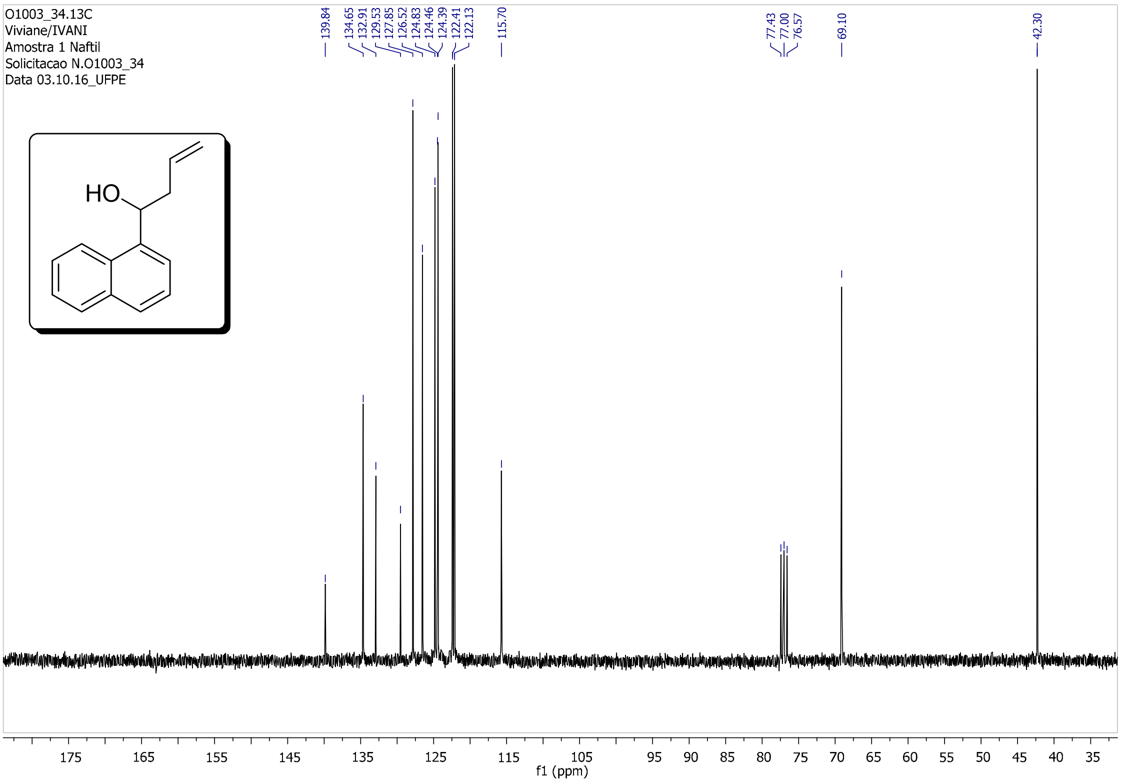

Figure S22. ^{13}C-NMR (300 MHz, $CDCl_3$) of 1-(naphtho-1-yl)but-3-en-1-ol (**3j**).

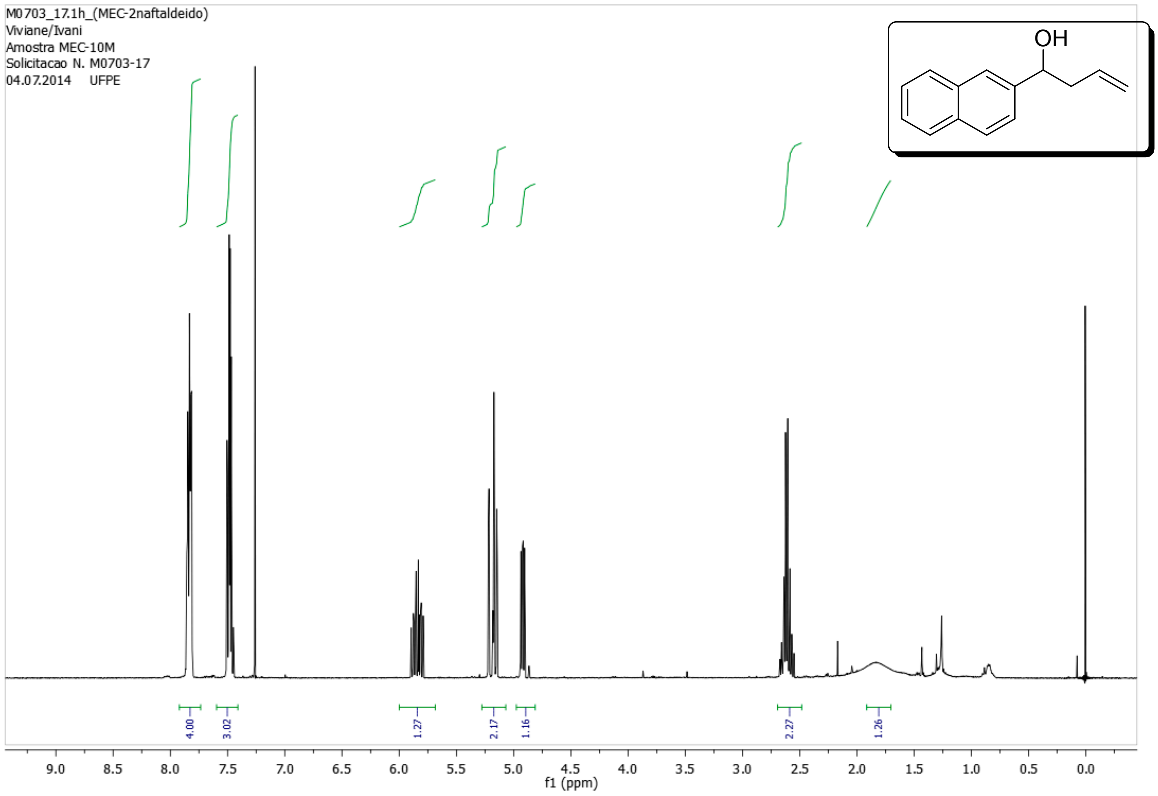

Figure S23. ^{1}H-NMR (300 MHz, $CDCl_3$) of 1-(naphtho-2-yl)but-3-en-1-ol (**3k**).

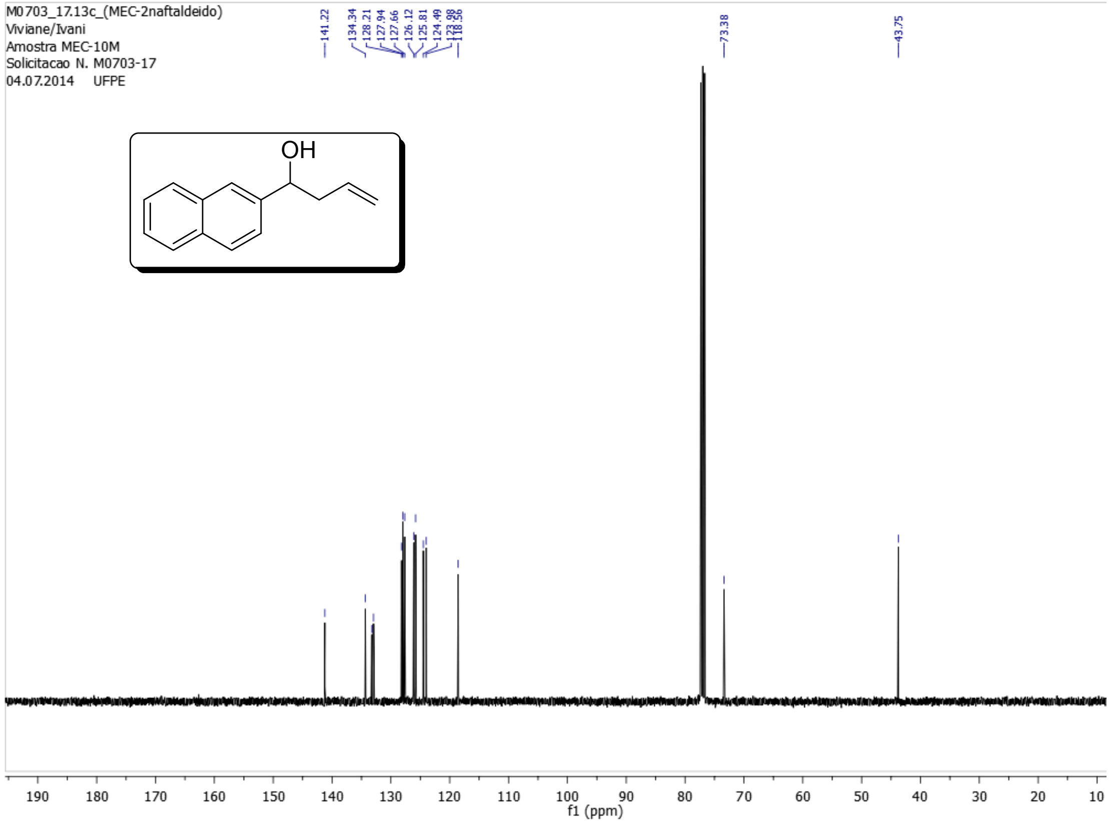

Figure S24. ^{13}C-NMR (75 MHz, $CDCl_3$) of 1-(naphtho-2-yl)but-3-en-1-ol (**3k**).

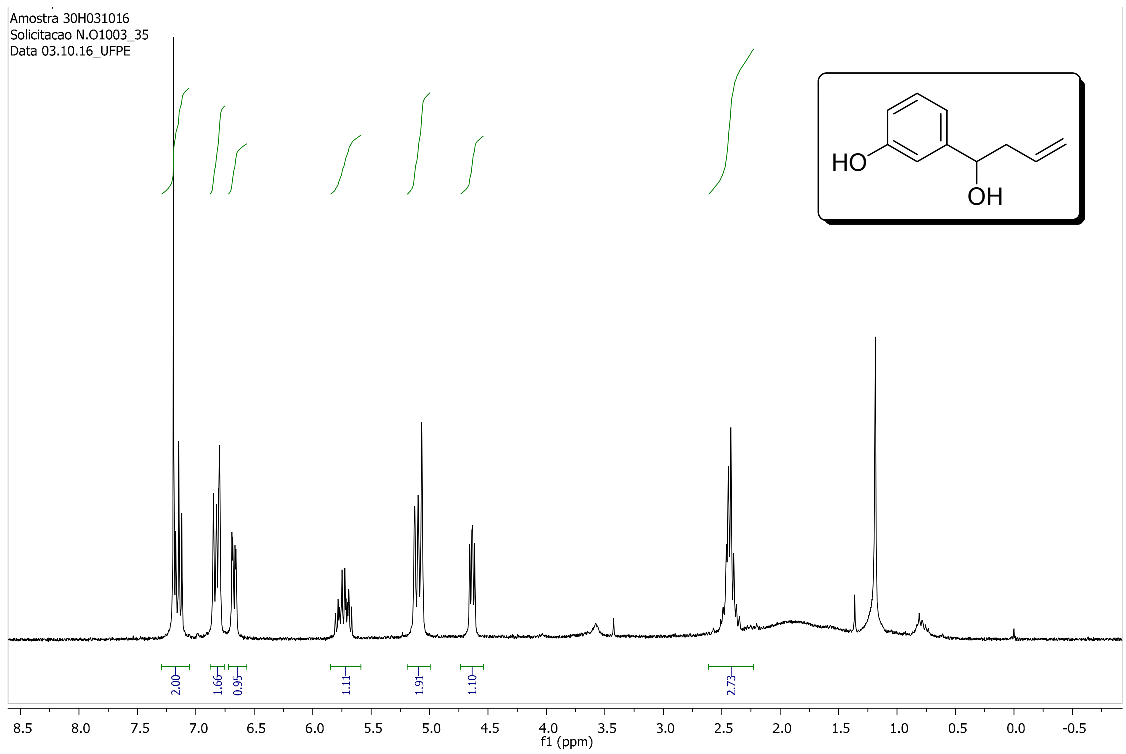

Figure S25. ^{1}H-NMR (100 MHz, $CDCl_3$) of 1-(3-Hydroxyphenyl)but-3-en-1-ol (**3l**).

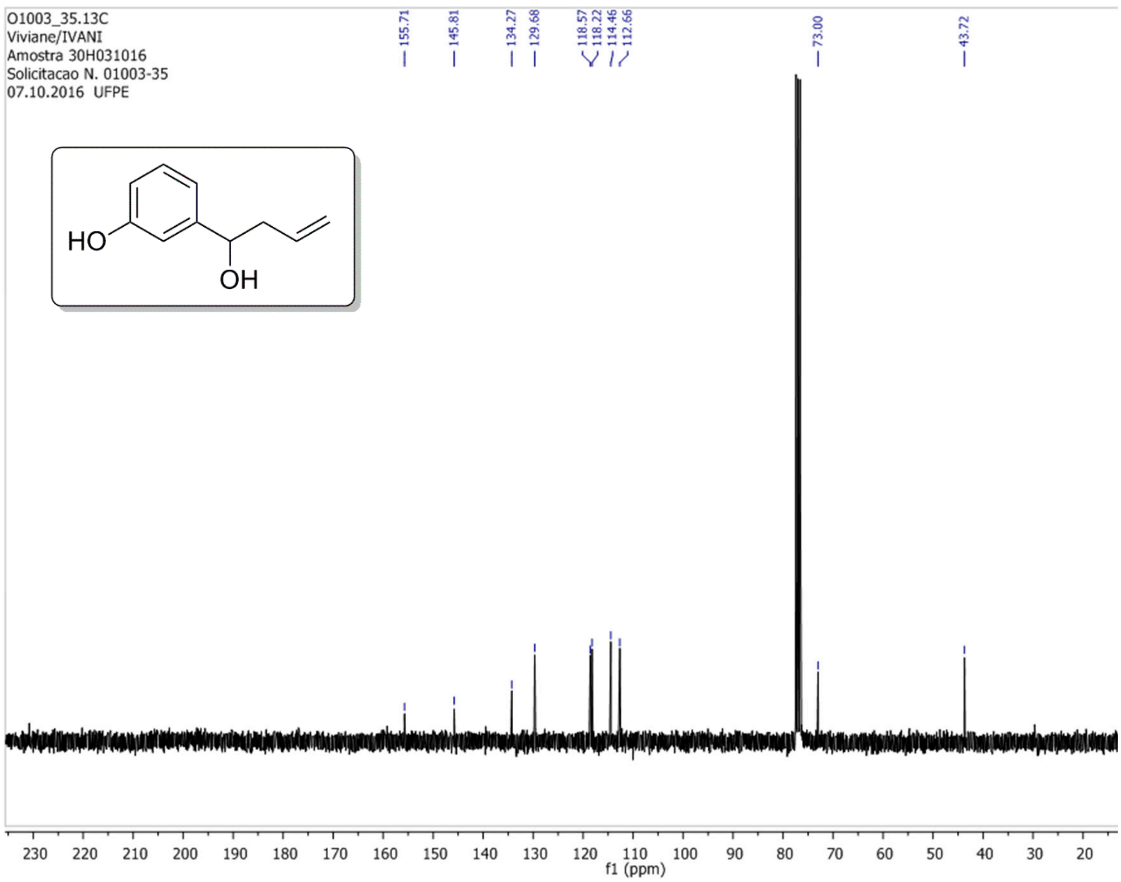

Figure S26. ^{13}C-NMR (75 MHz, $CDCl_3$) of 1-(3-Hydroxyphenyl)but-3-en-1-ol (**3l**).

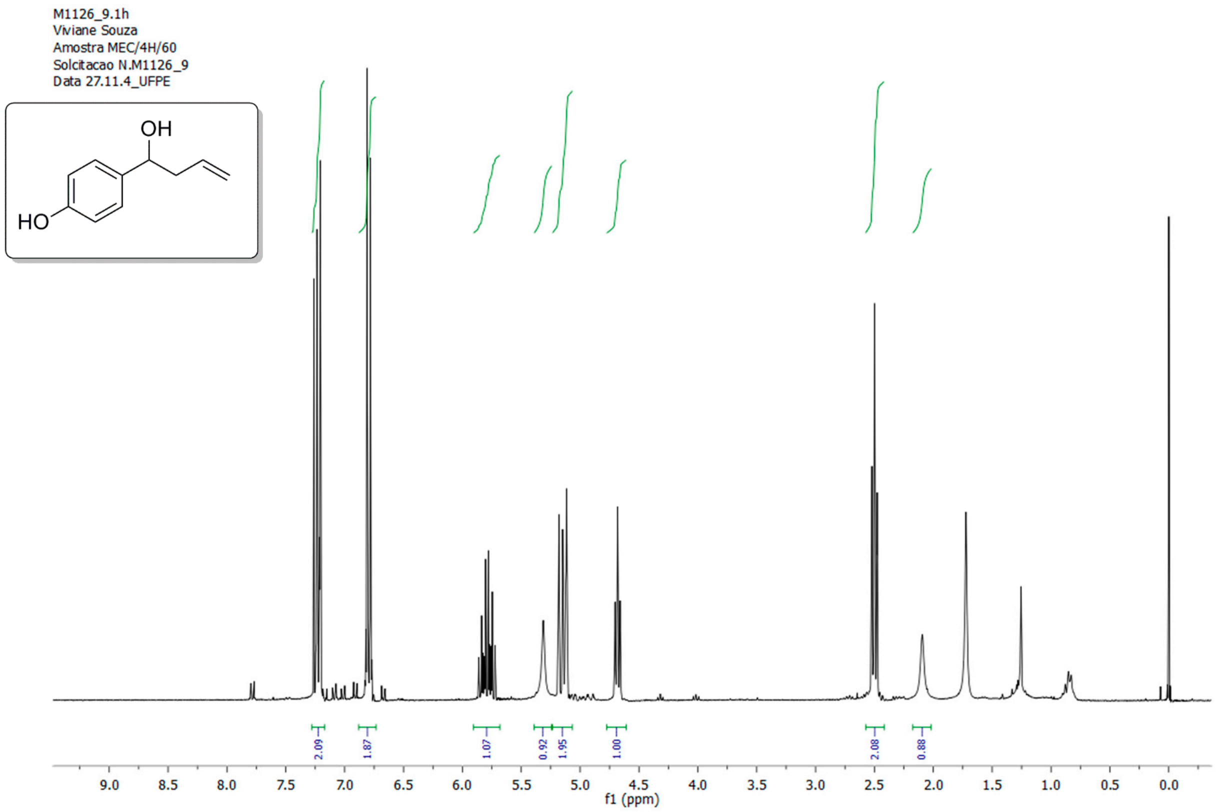

Figure S27. ^{1}H-NMR (300 MHz, $CDCl_3$) of 1-(4-Hydroxyphenyl)but-3-en-1-ol **(3m)**

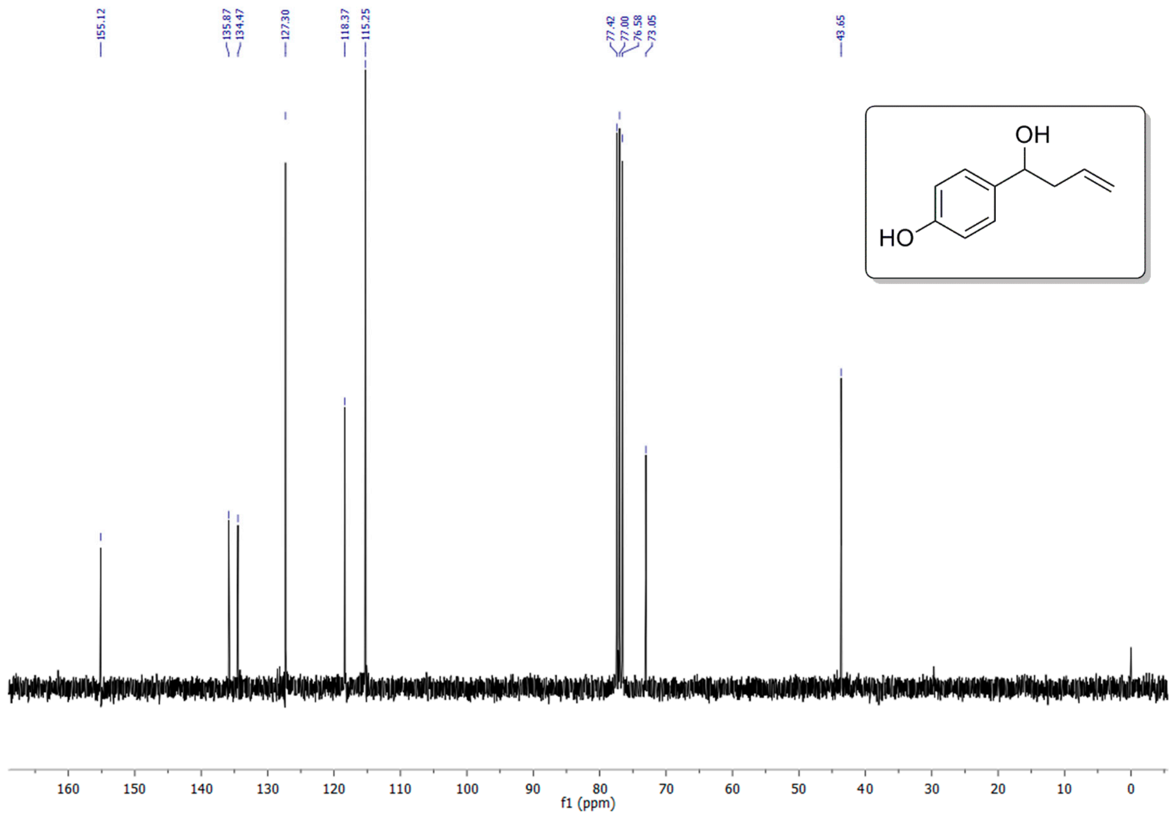

Figure S28. ^{13}C-NMR (75 MHz, $CDCl_3$) of 1-(4-Hydroxyphenyl)but-3-en-1-ol (**3m**).

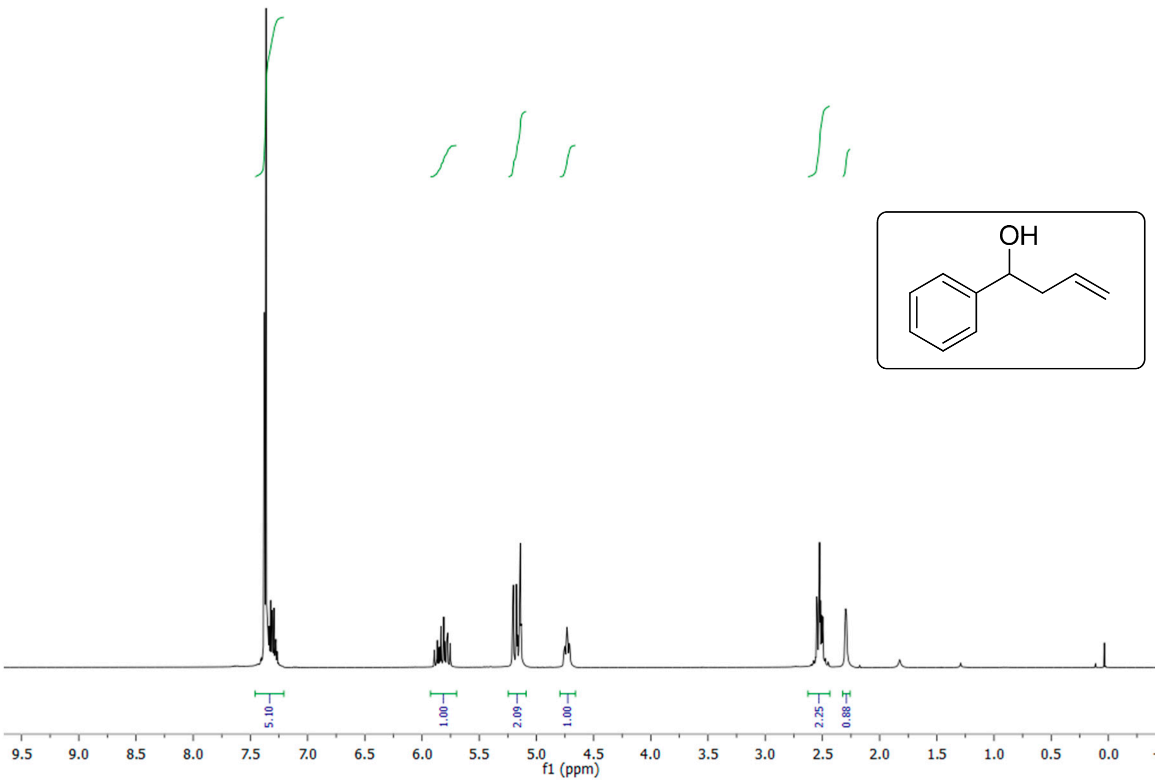

Figure S29. ^{1}H-NMR (300 MHz, $CDCl_3$) of 1-Phenylbut-3-en-1-ol (**3n**).

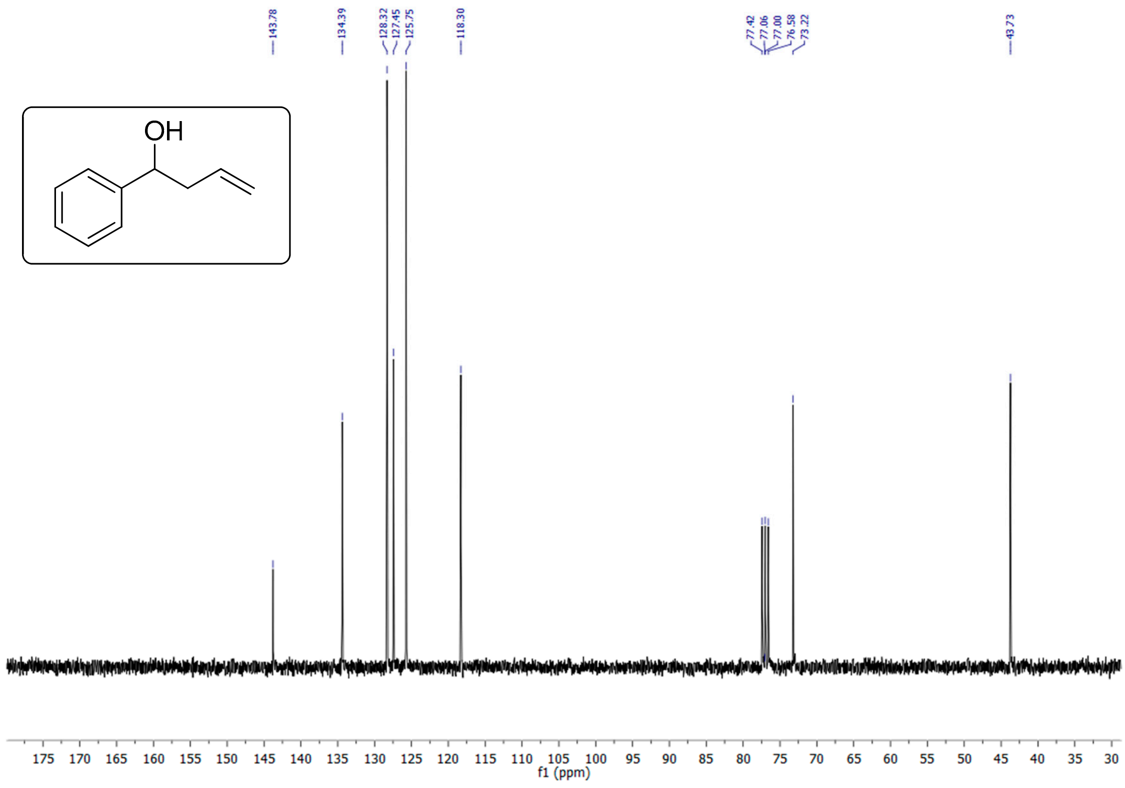

Figure S30. ^{13}C-NMR (75 MHz, $CDCl_3$) of 1-Phenylbut-3-en-1-ol (**3n**).

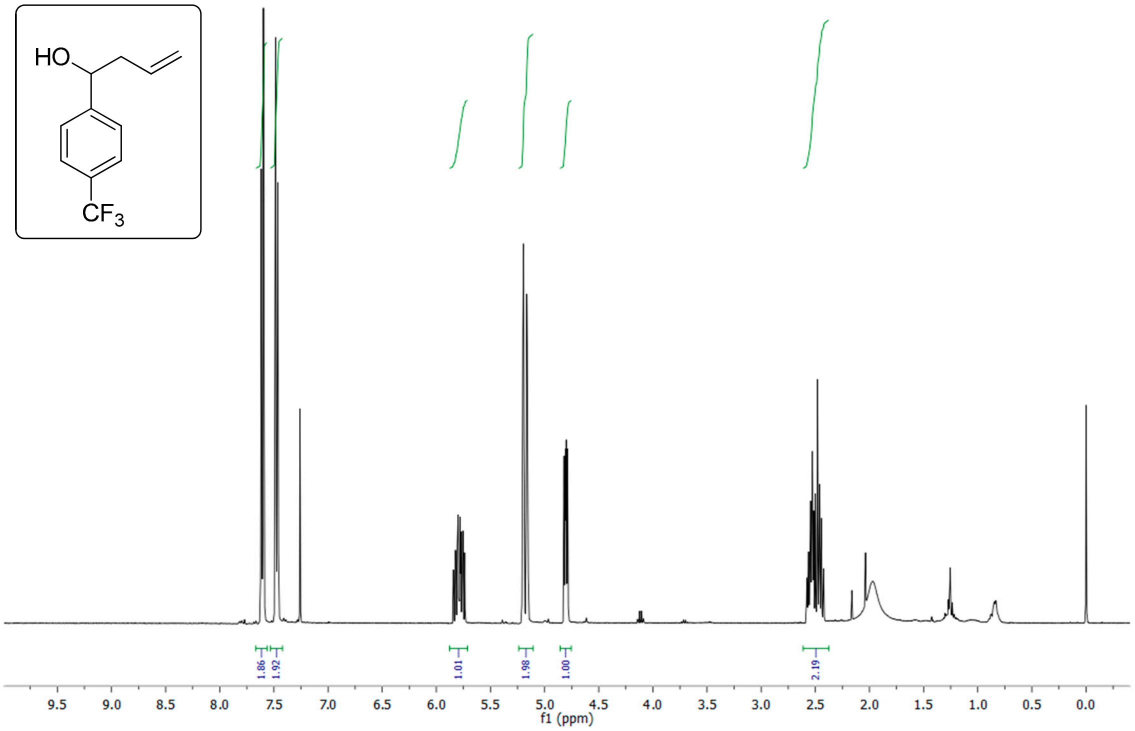

Figure S31. ^{1}H-NMR (400 MHz, $CDCl_3$) 1-(4-trifluoromethylphenyl)but-3-en-1-ol (**3o**).

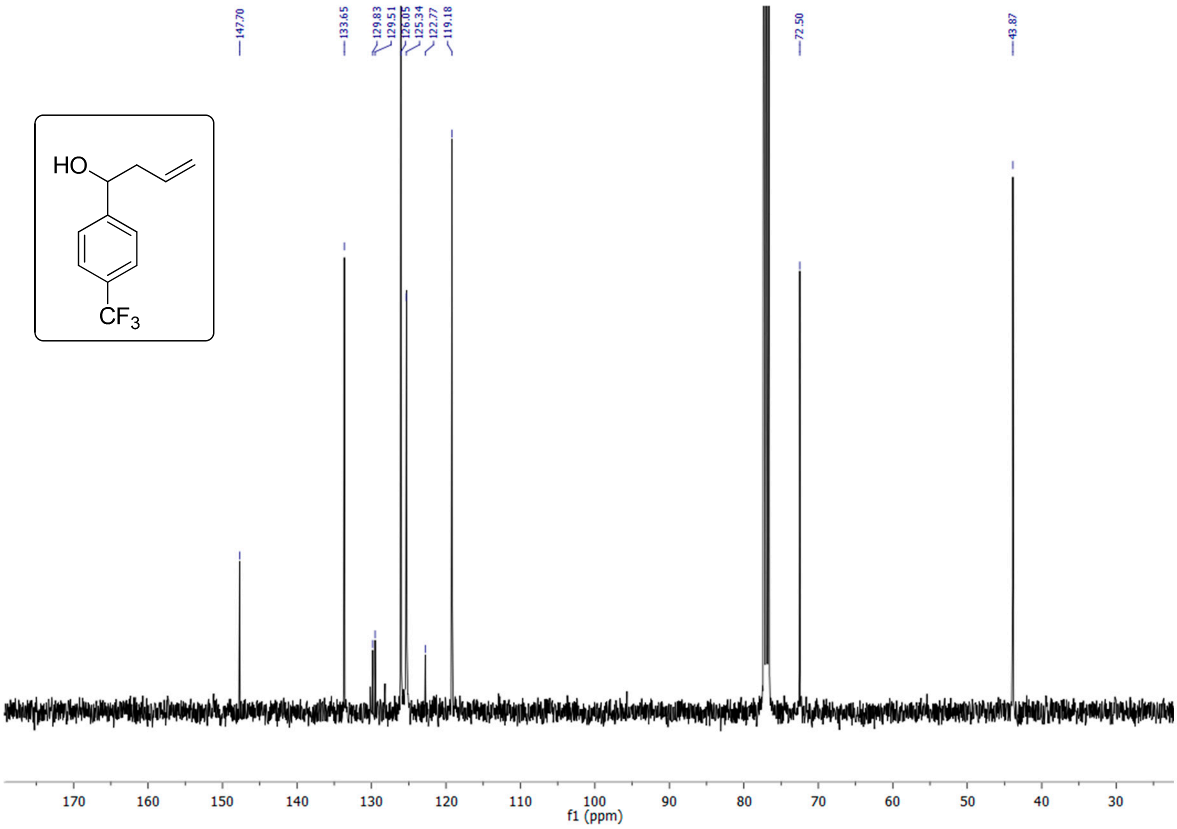

Figure S32. ^{13}C-NMR (100 MHz, $CDCl_3$) 1-(4-trifluoromethylphenyl)but-3-en-1-ol (**3o**).

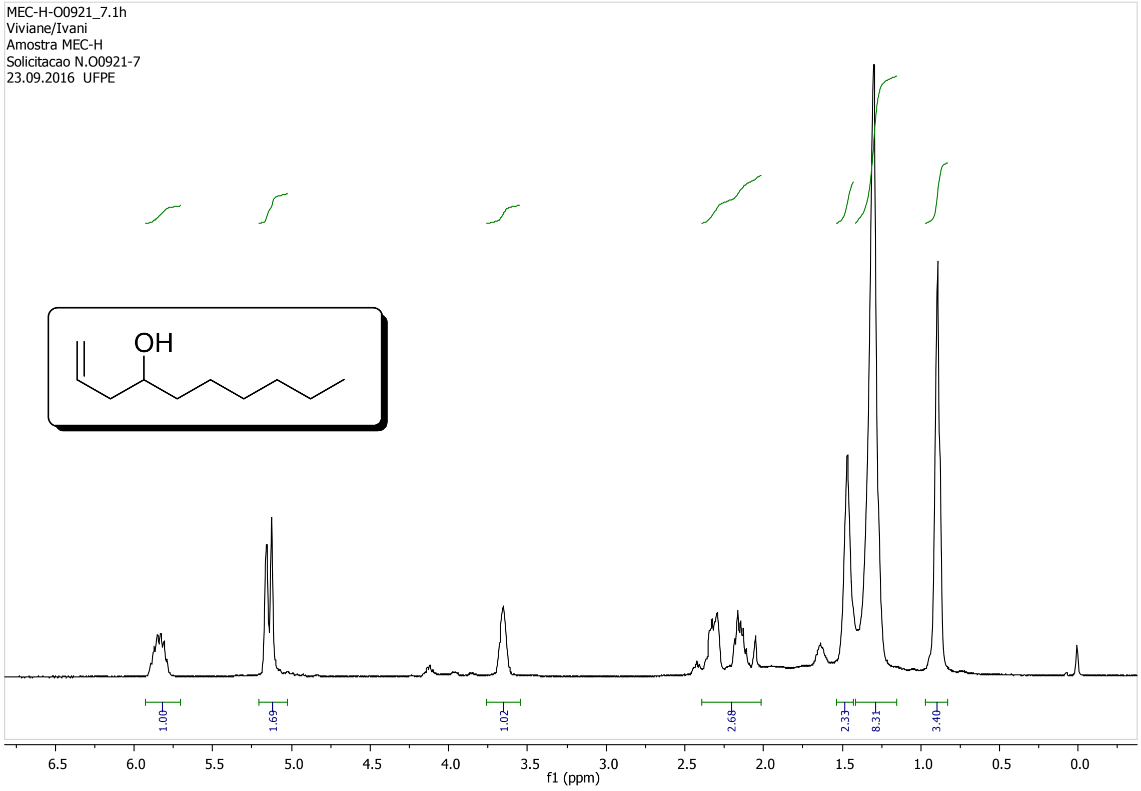

Figure S33. ^{1}H-NMR (300 MHz, $CDCl_3$) of Dec-1-en-4-ol (**3p**).

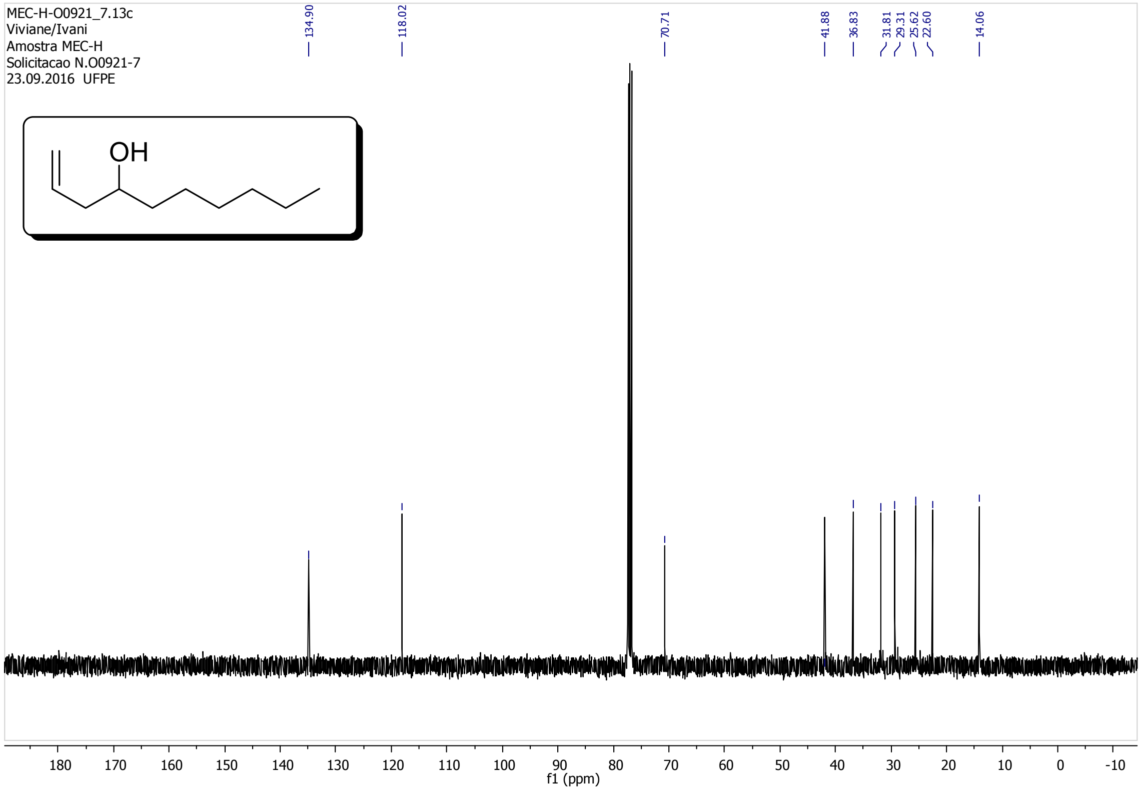

Figure S34. ^{13}C-NMR (75 MHz, $CDCl_3$) of Dec-1-en-4-ol (**3p**).

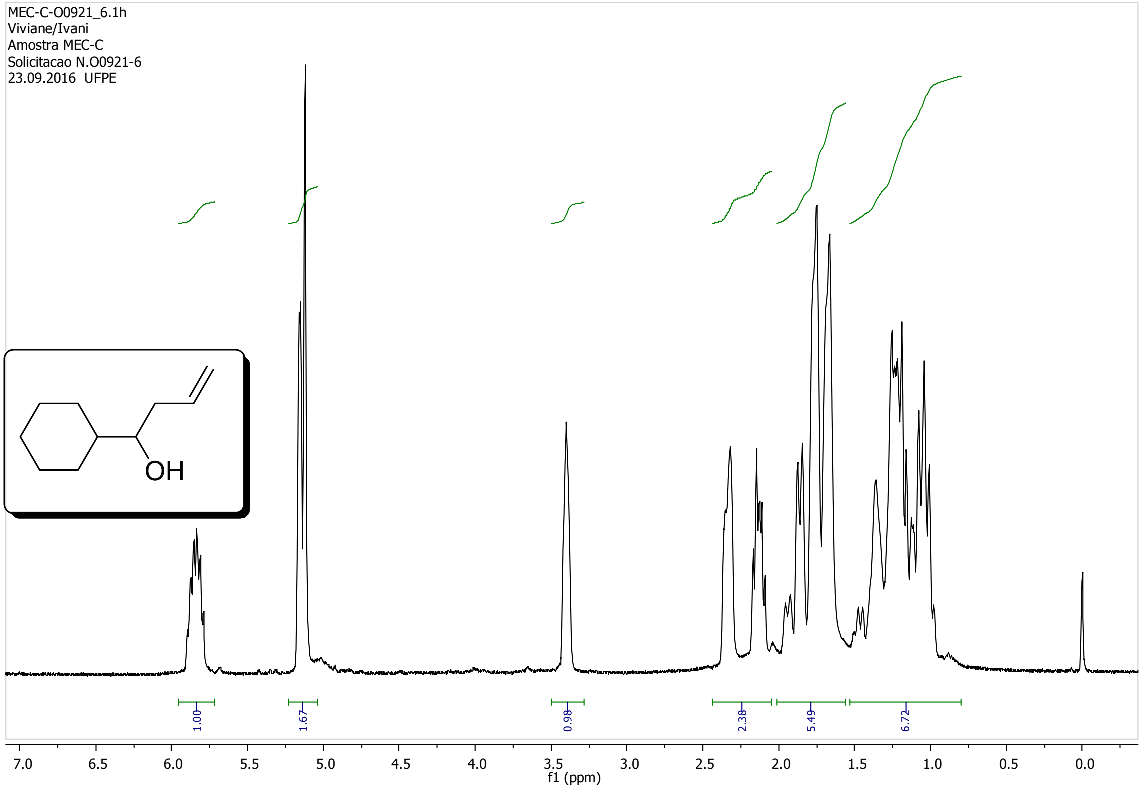

Figure S35. ^{1}H-NMR (300 MHz, $CDCl_3$) of 1-Cyclohexylbut-3-en-1-ol (**3q**).

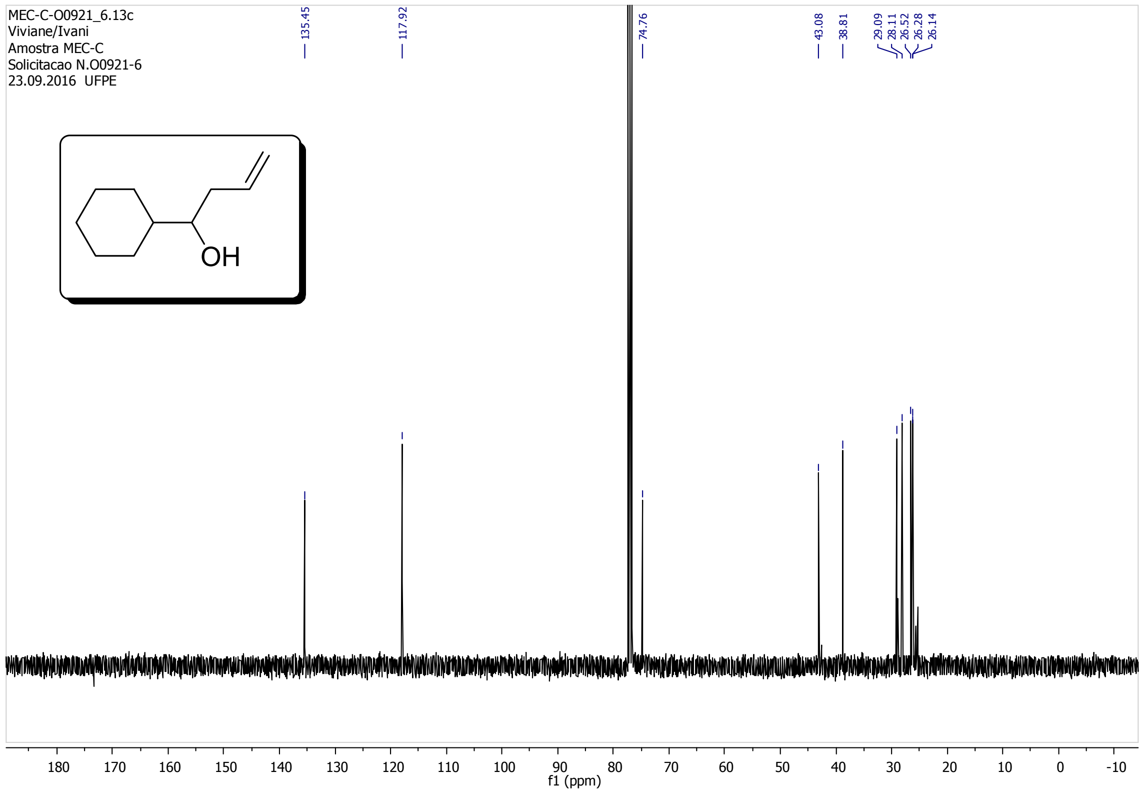

Figure S36. ^{13}C-NMR (75 MHz, $CDCl_3$) of 1-Cyclohexylbut-3-en-1-ol (**3q**).

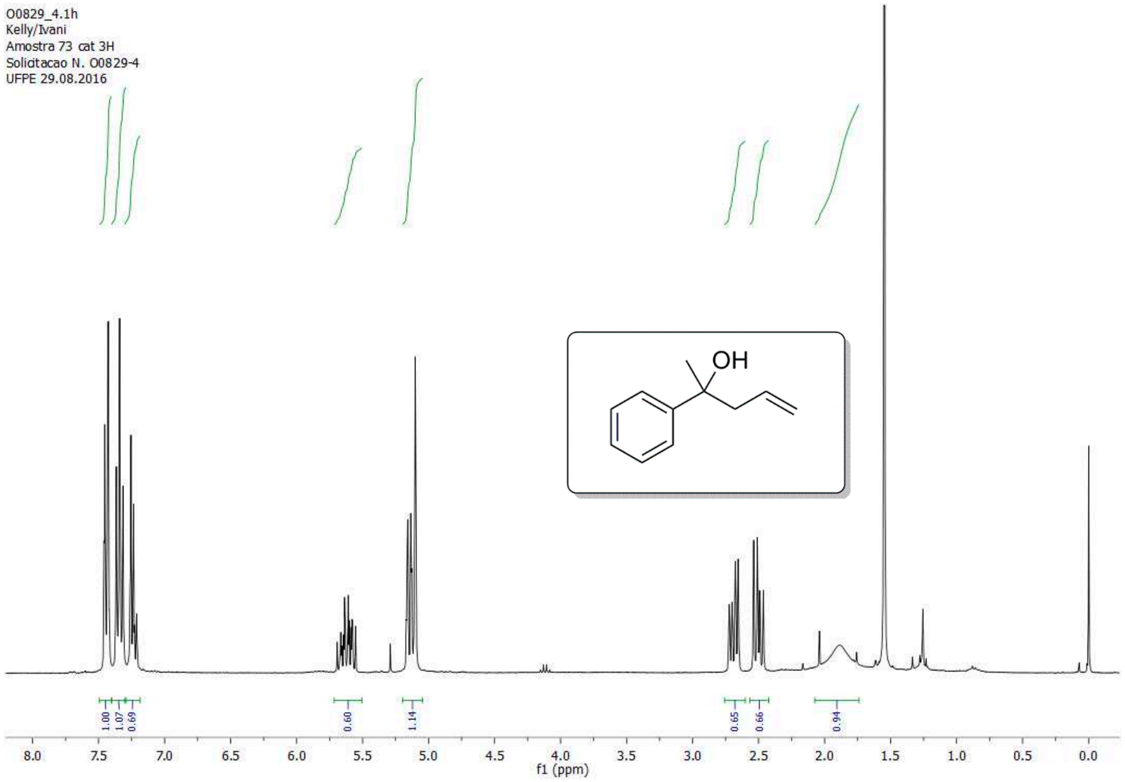

Figure S37. ^{1}H-NMR (300 MHz, $CDCl_3$) 2-phenylpent-4-en-2-ol (**5a**).

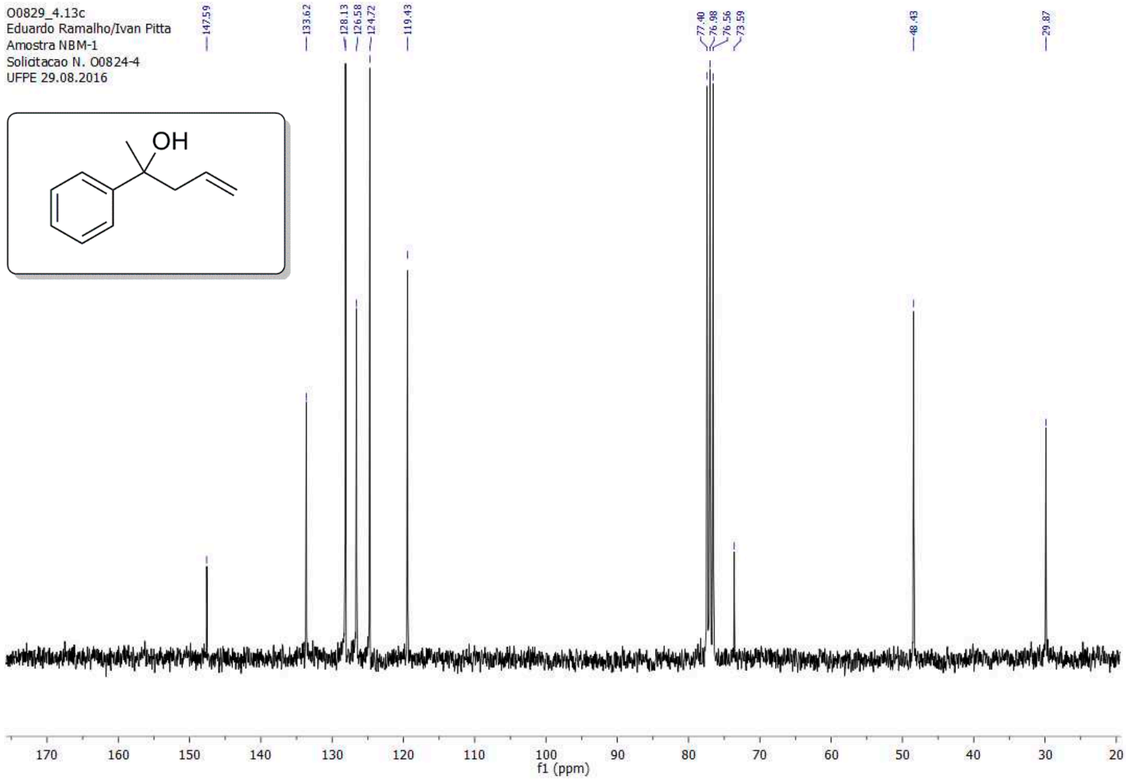

Figure S38. ^{13}C-NMR (75 MHz, $CDCl_3$) 2-phenylpent-4-en-2-ol (**5a**).

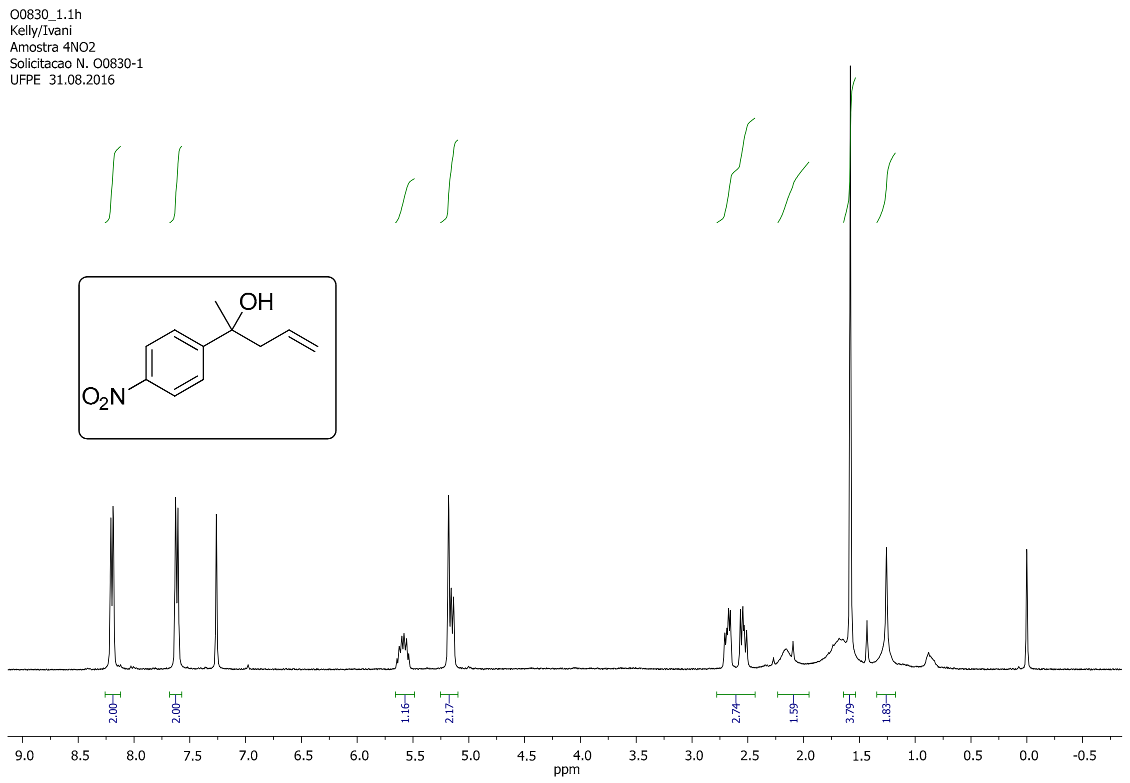

Figure S39. ^{1}H-NMR (400 MHz, $CDCl_3$) of 2-(4-nitrophenyl)pent-4-en-2-ol (**5b**).

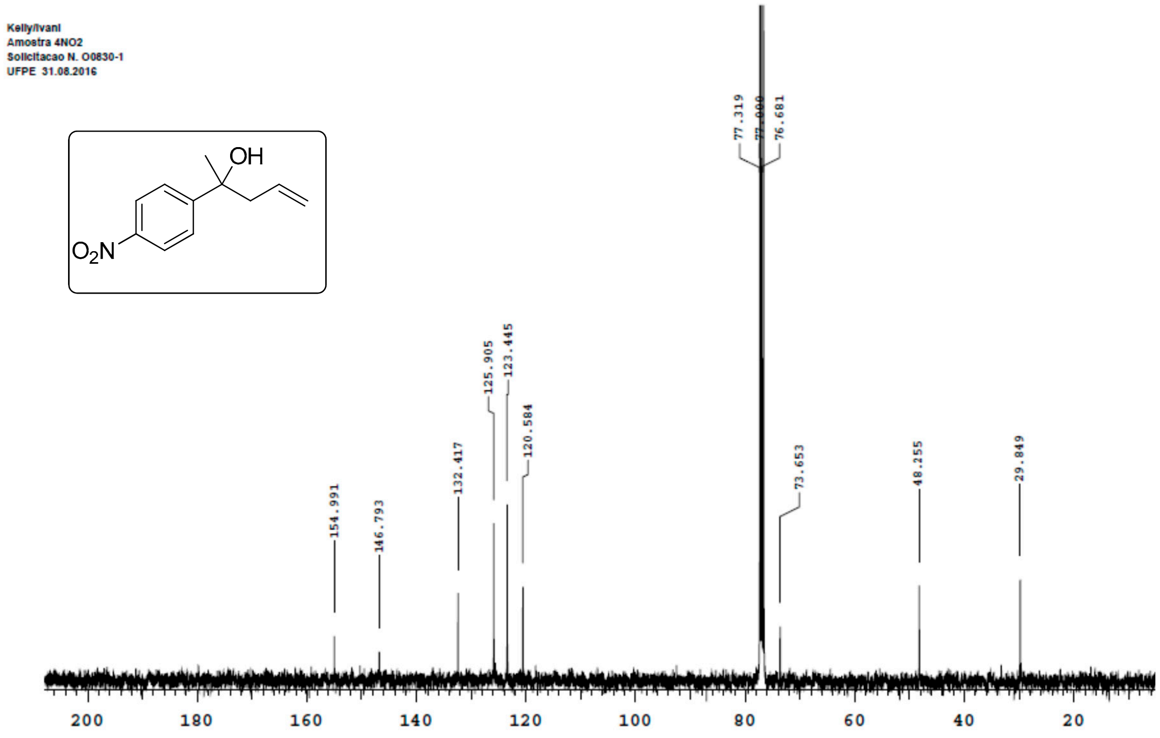

Figure S40. ^{13}C-NMR (100 MHz, $CDCl_3$) of 2-(4-nitrophenyl)pent-4-en-2-ol (**5b**).

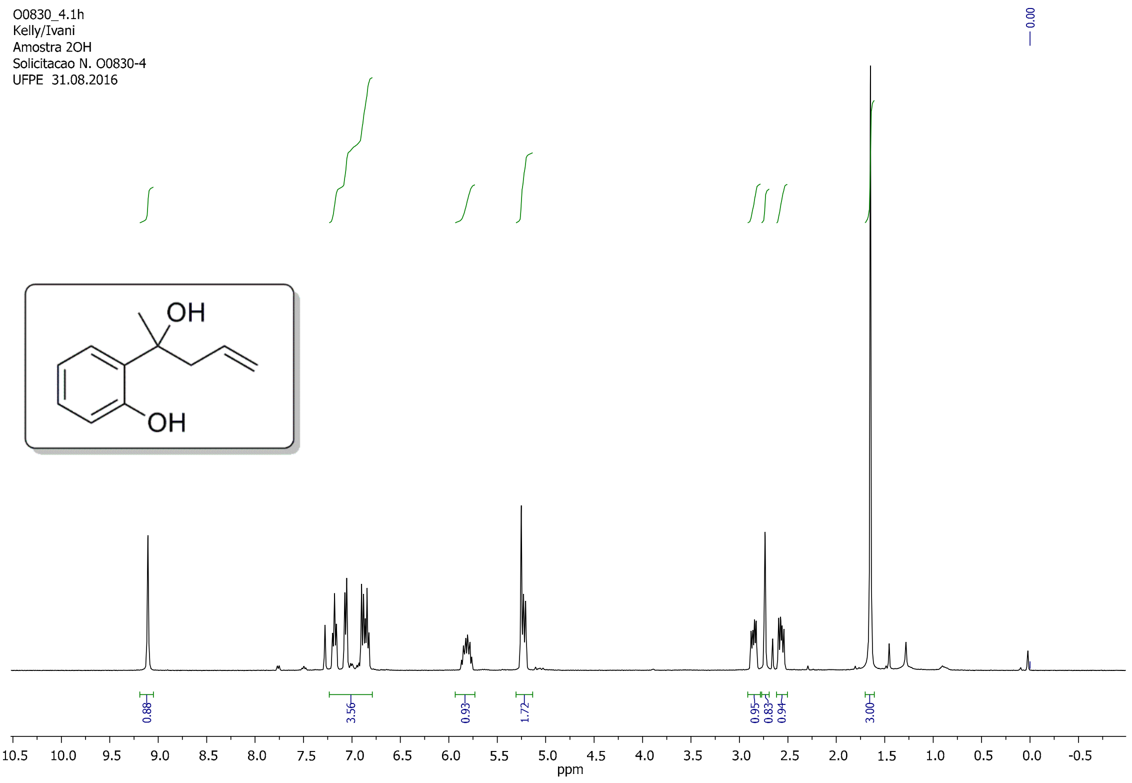

Figure S41. ^{1}H-NMR (400 MHz, $CDCl_3$) of 2-(2-Hydroxyphenyl)pent-4-en-2-ol (**5c**).

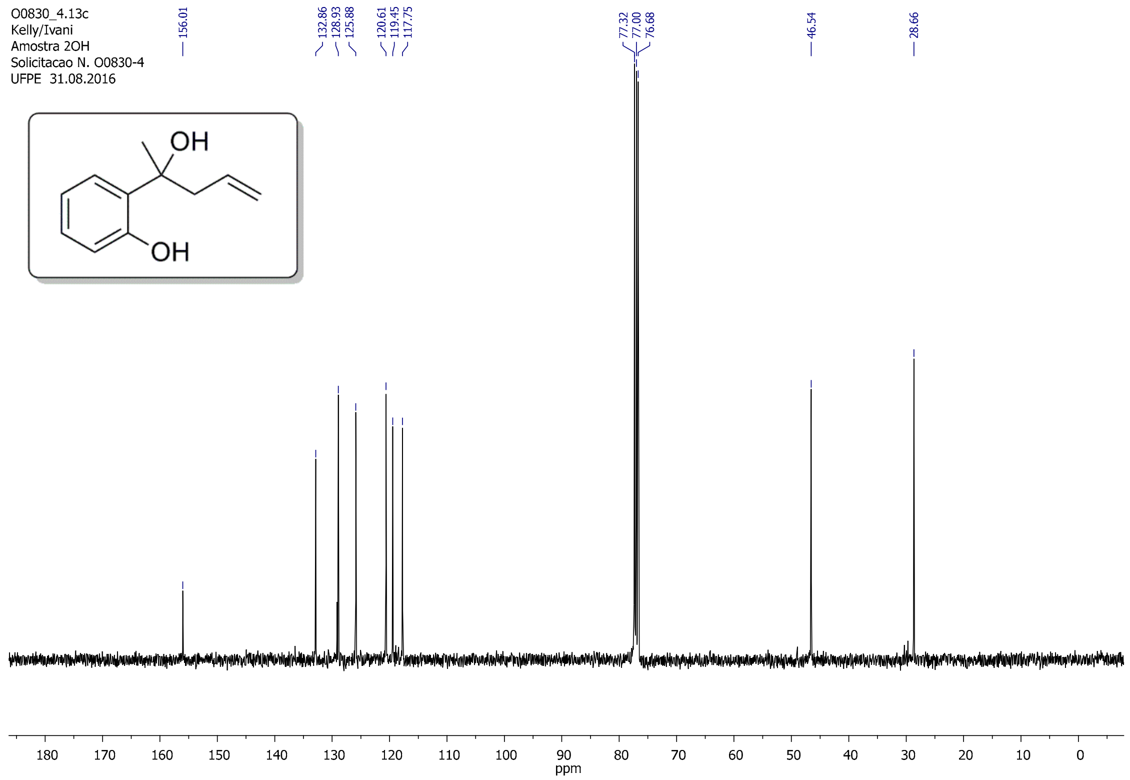

Figure S42. ^{13}C-NMR (100 MHz, $CDCl_3$) of 2-(2-Hydroxyphenyl)pent-4-en-2-ol (**5c**).

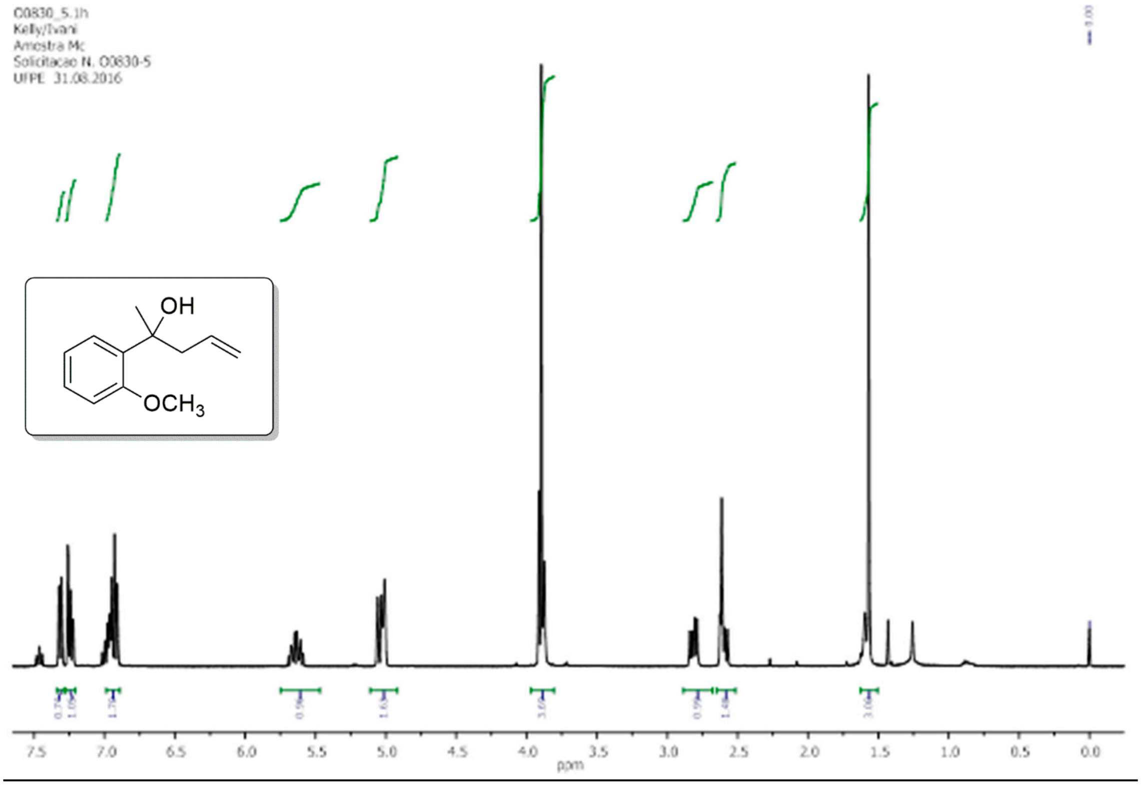

Figure S43. ^{1}H-NMR (400 MHz, $CDCl_3$) of 2-(2-methoxyphenyl)pent-4-en-2-ol (**5d**).

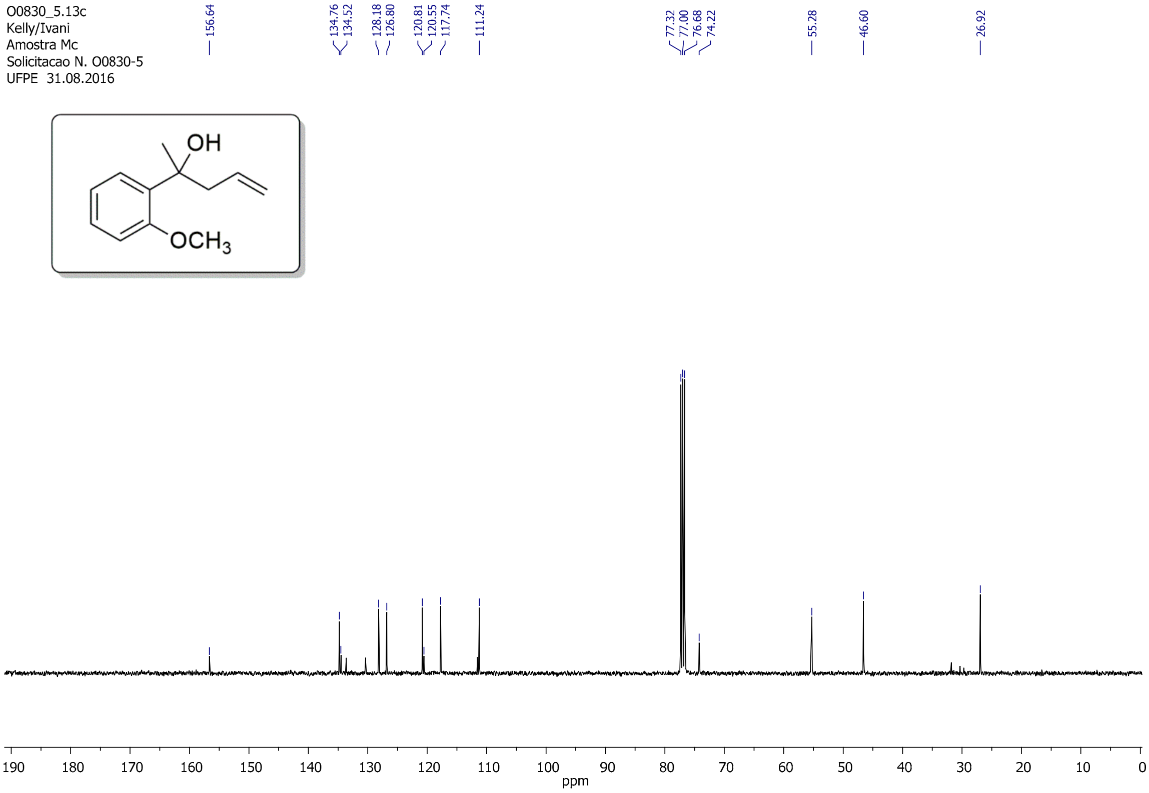

Figure S44. ^{13}C-NMR (100 MHz, $CDCl_3$) of 2-(2-methoxyphenyl)pent-4-en-2-ol (**5d**).

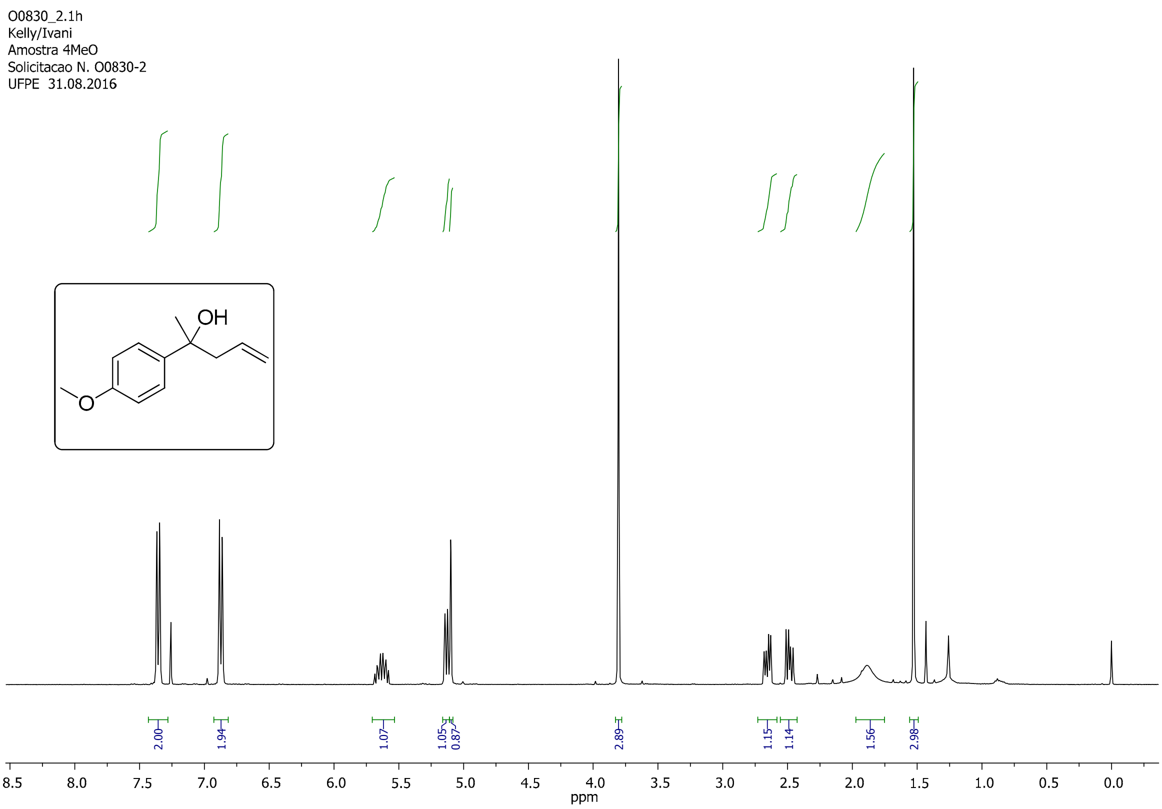

Figure S45. ^{1}H-NMR (400 MHz, $CDCl_3$) of 2-(4-methoxyphenyl)pent-4-en-2-ol (**5e**).

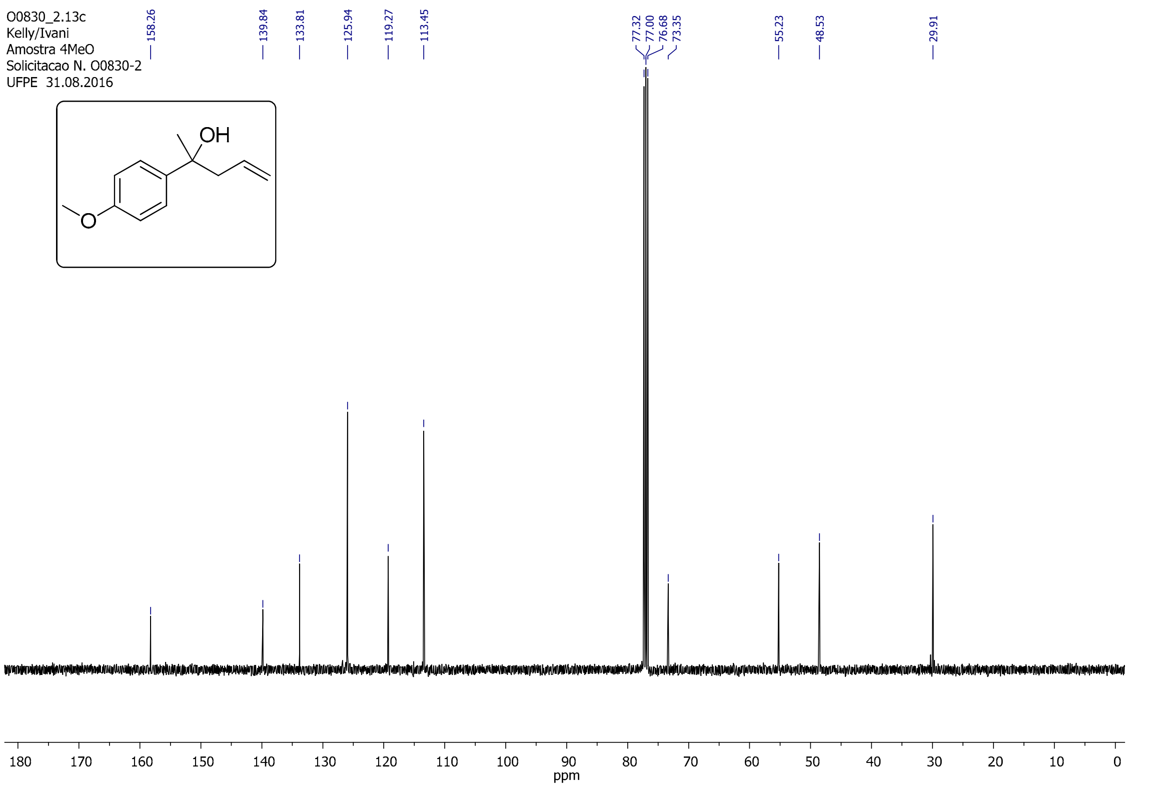

Figure S46. ^{13}C-NMR (100 MHz, $CDCl_3$) of 2-(4-methoxyphenyl)pent-4-en-2-ol (**5e**).

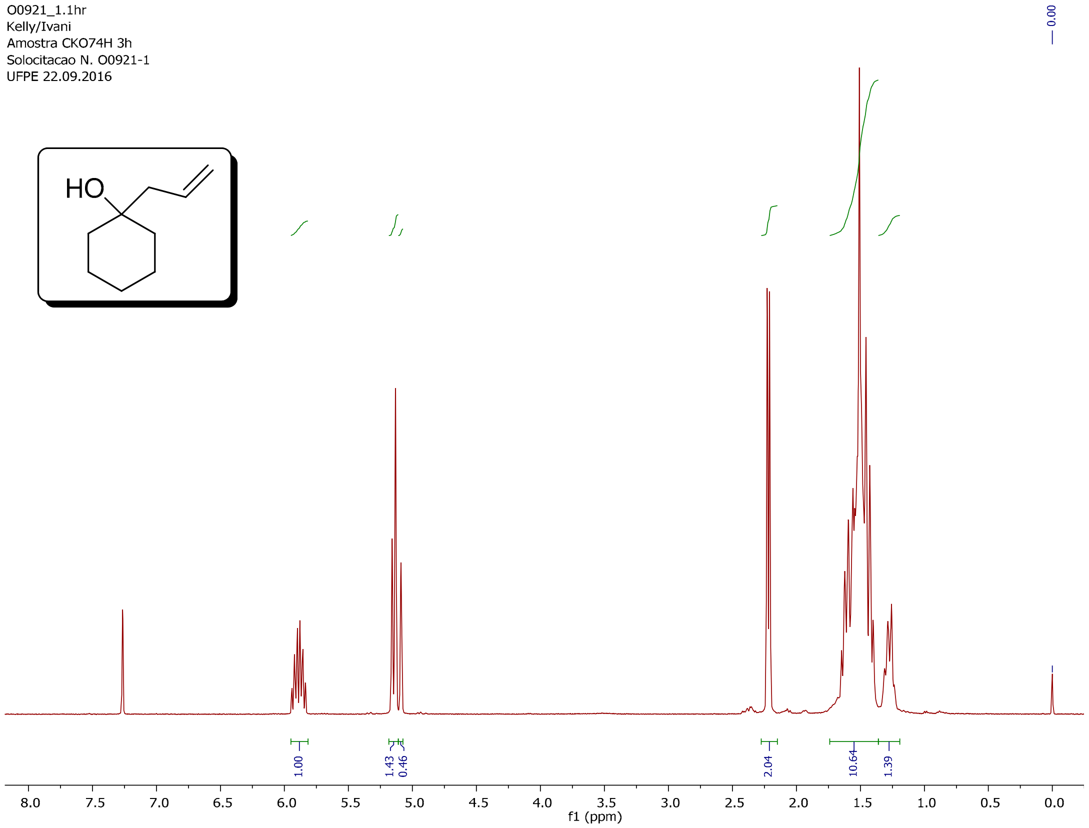

Figure S47. ^{1}H-NMR (400 MHz, $CDCl_3$) of 1-allylcyclohexanol (**5f**).

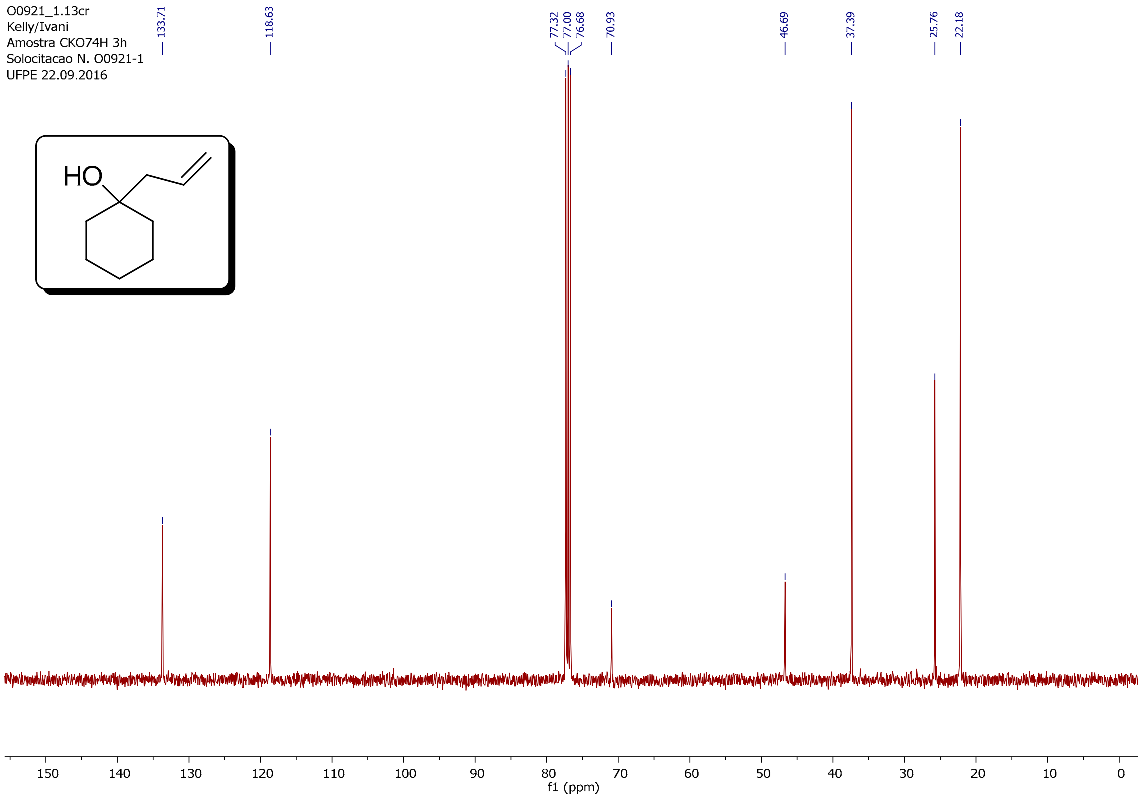

Figure S48. ^{13}C-NMR (100 MHz, $CDCl_3$) of 1-allylcyclohexanol (**5f**).

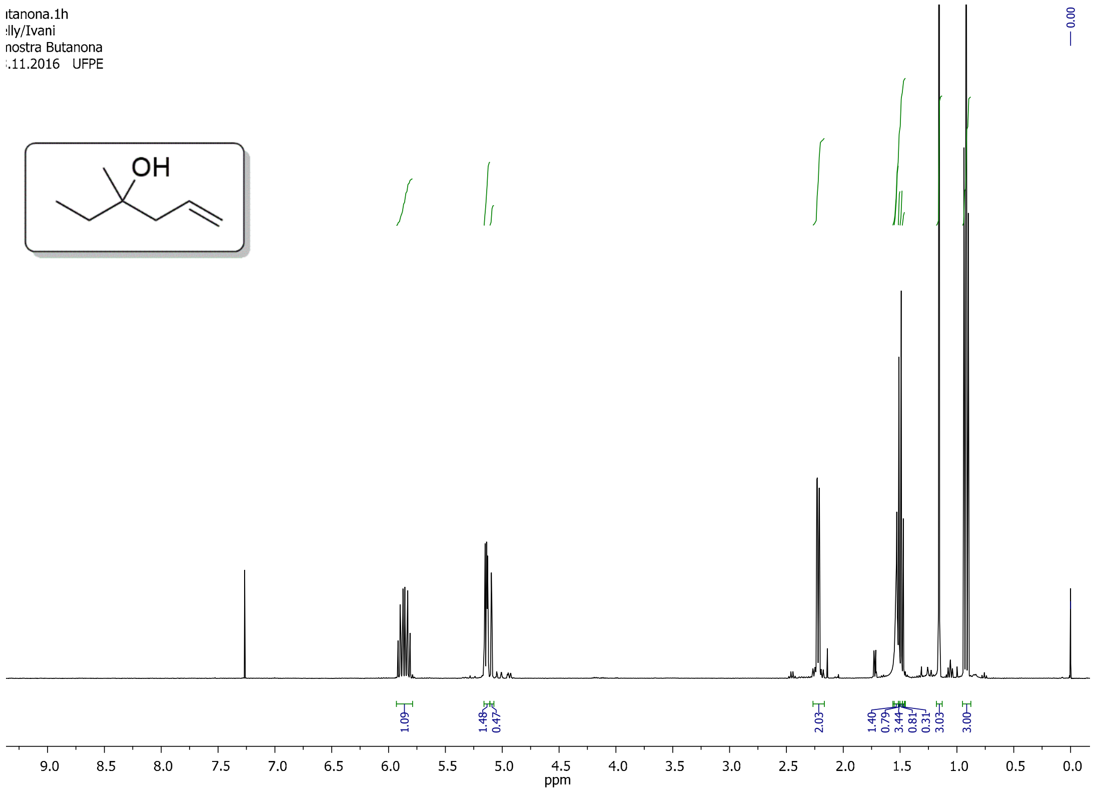

Figure S49. ^{1}H-NMR (400 MHz, $CDCl_3$) of 3-methylhex-5-en-3-ol (**5g**).

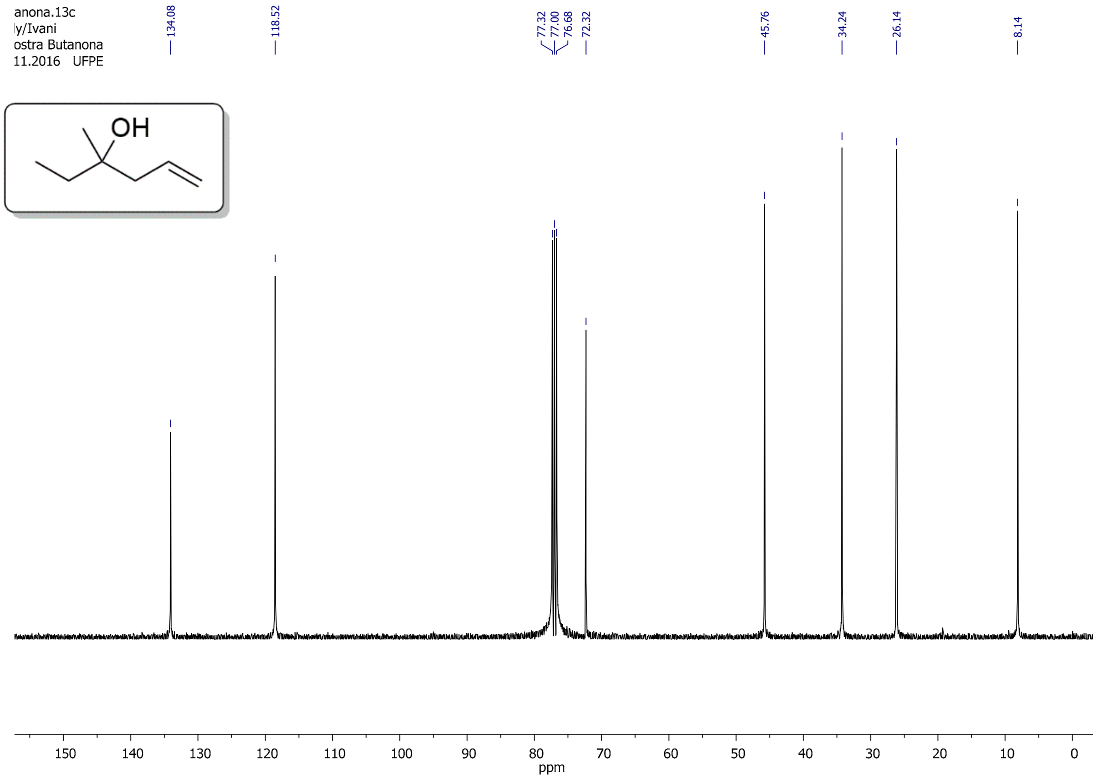

Figure S50. ^{13}C-NMR (100 MHz, $CDCl_3$) 3-methylhex-5-en-3-ol (**5g**).
